# Effect of VCP modulators on gene expression profiles of retinal ganglion cells in an acute injury mouse model

**DOI:** 10.1038/s41598-020-61160-6

**Published:** 2020-03-06

**Authors:** Tomoko Hasegawa, Hanako Ohashi Ikeda, Norimoto Gotoh, Kei Iida, Sachiko Iwai, Noriko Nakano, Akira Kakizuka, Akitaka Tsujikawa

**Affiliations:** 1 Department of Ophthalmology and Visual Sciences, Kyoto University Graduate School of Medicine, Kyoto, 606-8501 Japan; 2 Medical Research Support Center, Kyoto University Graduate School of Medicine, Kyoto, 606-8501 Japan; 3 Laboratory of Functional Biology, Kyoto University Graduate School of Biostudies & Solution Oriented Research for Science and Technology, Kyoto, 606-8501 Japan

**Keywords:** Drug discovery, Neuroscience, Eye diseases

## Abstract

In glaucoma, retinal ganglion cells are damaged, leading to the progressive constriction of the visual field. We have previously shown that the valosin-containing protein (VCP) modulators, Kyoto University Substance (KUS)121 and KUS187, prevent the death of retinal ganglion cells in animal models of glaucoma, including the one generated by N-methyl-D-aspartate (NMDA)-induced neurotoxicity. KUSs appeared to avert endoplasmic reticulum (ER) stress by maintaining ATP levels, resulting in the protection of ganglion cells from cell death. To further elucidate the protective mechanisms of KUSs, we examined gene expression profiles in affected ganglion cells. We first injected KUS-treated mice with NMDA and then isolated the affected retinal ganglion cells using fluorescence-activated cell sorting. Gene expression in the cells was quantified using a next-generation sequencer. Resultantly, we found that KUS121 upregulated several genes involved in energy metabolism. In addition, we observed the upregulation of *Zfp667*, which has been reported to suppress apoptosis-related genes and prevent cell death. These results further support the suitability of KUS121 as a therapeutic drug in protecting retinal ganglion cells in ophthalmic disorders, such as glaucoma.

## Introduction

Glaucoma is one of the leading causes of blindness around the world^1–4^. In this disease, retinal ganglion cells are damaged followed by progressive visual field constriction^5,6^. The most commonly used evidenced treatment for glaucoma involves reducing intraocular pressure with drugs or surgery, and it effectively slows the deterioration of visual function^7–9^. While high intraocular pressure and age are known risk factors for glaucoma progression^10^, the possible involvement of myopia and blood flow impairment remain controversial^10–14^, thus, the glaucoma pathologies are not fully understood.

We have previously synthesised novel compounds, Kyoto University Substances (KUSs), which mitigate cellular ATP reduction by modulating the ATPase activity of valosin-containing protein (VCP)^15^, the most abundant soluble ATPase in the cell. KUSs prevented ATP depletion, endoplasmic reticulum (ER) stress, and consequently cell death in cultured cells. KUSs consistently suppressed retinal neuronal cell death in animal models of ocular diseases, such as retinitis pigmentosa^15,16^, glaucoma^17^, and central retinal artery occlusion^18^.

Intravitreous injection of N-methyl-D-aspartate (NMDA) induces neurotoxicity mainly in retinal ganglion cells^19^. Administration of KUSs prevented the decrease of the retinal ganglion cells and nerve fibers, in the acute retinal injury model induced by NMDA^17^. In addition to the suppression of the decrease of ATP levels, we aimed to clarify the potential involvement of cellular genes by the KUS treatment. Towards this end, isolation and collection of retinal ganglion cells is needed because they consist of only a small proportion of retinal cells^20^. A two-step immunopanning and magnetic separation^21–24^, or combined immunopanning-magnetic separation^25^ have been used to isolate retinal ganglion cells previously^26–28^. The use of fluorescence-activated cell sorting (FACS) is another way to collect the retinal ganglion cells. These methods allow us to isolate fresh ganglion cells for RNA analyses, which faithfully reflect the *in vivo* state. Thy1-CFP transgenic mice^29^ (referred hereafter as Thy1-CFP mice) express cyan fluorescent protein (CFP) in the retinal ganglion cells^30^ under the Thy1 promoter^31,32^, which enabled us to purify retinal ganglion cells by FACS. Next, we used next-generation sequencing technologies^33^ to compare gene expression profiles between with and without KUS treatments.

## Results

### mRNA expression of key genes was significantly altered 4 h after NMDA injection

**Figure 1:**
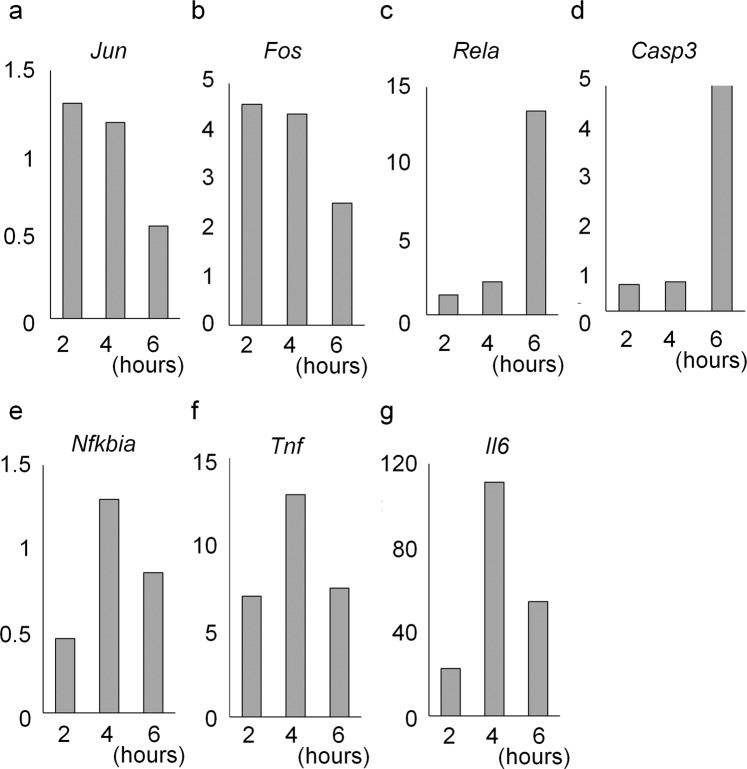
Relative mRNA expression at different time points after intravitreal N-methyl-D-aspartate (NMDA) injection. Neural retinas were analysed 2, 4 and 6 h after intravitreal NMDA (5 nmol) injection. The relative expression levels of (**a**) jun proto-oncogene (*Jun*), (**b**) FBJ osteosarcoma oncogene (*Fos*), (**c**) v-rel reticuloendotheliosis viral oncogene homolog A (*Rela*), (**d**) capsase-3 (*Casp3*), (**e**) nuclear factor of kappa light polypeptide gene enhancer in B cells inhibitor, alpha (*Nfkbia*), (**f**) tumor necrosis factor (*Tnf*) and (**g**) interleukin 6 (*Il6*) mRNA were analysed by qRT-PCR. The ratios of mRNA expression of each gene to that of actin were calculated. The ratios to actin at 2 h, 4 h and 6 h were divided by those at 0 h.

To decide the timing for evaluation of gene expression after intravitreous NMDA injection, the mRNA levels of 18 genes, of which some were reported to be upregulated after NMDA injection and some could be influenced by administration of KUSs, were analysed using quantitative reverse transcription polymerase chain reaction (qRT-PCR). The former were v-rel reticuloendotheliosis viral oncogene homolog A (*Rela*), caspase 3 (*Casp3*), nuclear factor of kappa light polypeptide gene enhancer in B cells inhibitor - alpha (*Nfkbia*), interleukin 6 (*Il6*), FBJ osteosarcoma oncogene (*Fos*), mitogen-activated protein kinase (*Mapk*)1, *Mapk3*, *Mapk*1*0*, jun proto-oncogene (*Jun*), tumor necrosis factor (*Tnf*), and high mobility group box 1 (*Hmgb*1) and the latter were serine/threonine-protein kinase (*Akt*)*1*, *Akt2*, mitochondrial fission 1 (*Fis1*), mitofusin (*Mfn*)*1*, *Mfn2*, dynamin 1-like (*Dnm1l*), optic atrophy 1 (*Opa1*). *Jun* and *Fos*, which were reported to be immediately upregulated following external stimuli^34^ increased 2 h after the NMDA injection (Fig. 1). Expression of *Rela* and *Casp3* (a downstream effector of apoptosis) increased prominently 6 h after the injection (Fig. 1). In contrast, expression of the other 14 genes including *Nfkbia*, *Tnf*, and *Il-6* increased notably 4 h after the NMDA injection (Fig. 1). Therefore, we decided to evaluate the effect of KUSs on the gene expression profiles 4 h after the NMDA injection.

### Purification of retinal ganglion cells by FACS

**Figure 2:**
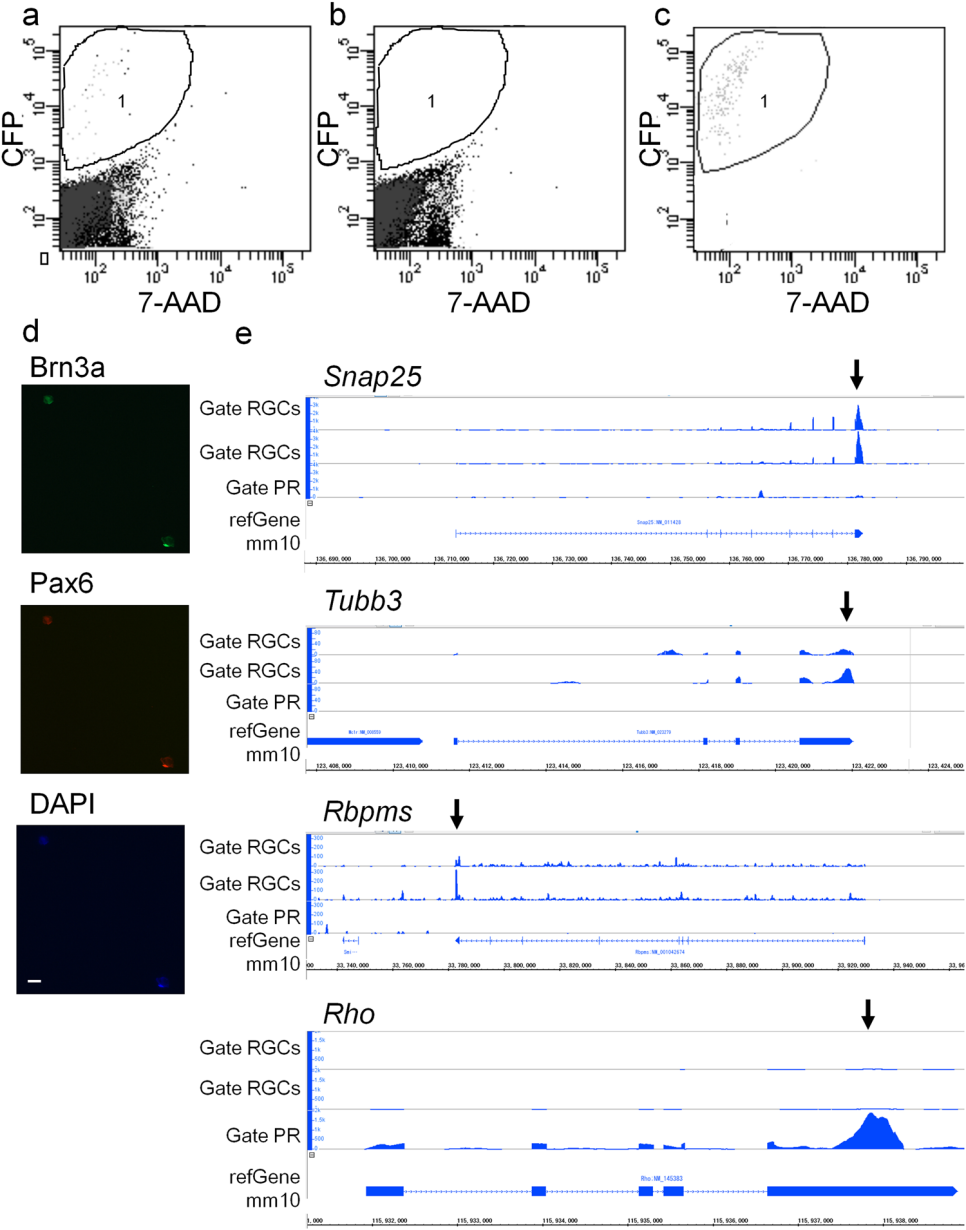
Analysis of dissociated retinal cells and sorted cells by flow cytometry. Retinal cells were analysed using fluorescence-activated cell sorting. The x-axis shows fluorescent of PerCP-Cy5 to detect 7-Amino-Actinomycin D (7-AAD) fluorescence which labels dead cells and the y-axis shows fluorescence of AmCyan-A to detect cyan fluorescent protein (CFP) fluorescence. (**a**) Analysis of dissociated retinal cells of Thy1-CFP mice which manifest CFP in retinal ganglion cells. Cells which possess relatively strong CFP fluorescent without 7-AAD fluorescence, whose CFP fluorescence was considered not to be autofluorescence, were contained in the area 1 (0.04–0.12% of total cells). (**b**) Analysis of dissociated retinal cells of wild-type mice. No cells were contained in the area 1 (CFP-positive). (**c**) Re-analysis of the sorted cells by gate RGCs (see Method and Fig. S1). 88.9–93.4% of the sorted cells were included in area 1 (CFP-positive). (**d**) The sorted cells were stained with anti-brain-specific homeobox/POU domain protein 3A (Brn3a, green) and anti- paired box genes 6 (Pax6, red) antibodies. Nuclei were counter stained with 4’,6-diamidino-2-phenylindole (DAPI, blue). Scale bar: 50 µm. (**e**) mRNA expression of synaptosomal-associated protein 25 (*Snap25*), tubulin, beta 3 class III (*Tubb3*), RNA binding protein with multiple splicing (*Rbpms*) and rhodopsin (*Rho*) in cells sorted by gate RGCs or gate PR (see Methods and Supplementary, Fig. S1b) were visualised with the Integrated Genome Browser. *Snap25*, *Tubb3* and *Rbpms* were highly expressed in Gate RGCs and not in Gate PR while *Rho* was highly expressed in Gate PR and not in Gate RGCs (black arrows). RGC: retinal ganglion cell, PR: photoreceptors.

To study the effects of the KUSs on the affected cells, we first isolated the CFP expressing retinal ganglion cells from dissociated whole neural retina of Thy1-CFP mice using FACS. The CFP-positive cells were found to account for 0.04–0.12% of all retinal cells (Fig. 2a, area 1); no CFP-positive cells were observed in the retina of wild-type mice (Fig. 2b). Retinal cells expressing both brain-specific homeobox/POU domain protein 3A (Brn3a) and paired box protein (Pax) 6 are defined as retinal ganglion cells. By immunostaining the dissociated cells, we confirmed that Brn3a- and Pax6-positive retinal ganglion cells accounted for 0.01–0.12% of the total retinal cells. The percentage of the retinal ganglion cells estimated with the immunocytochemical analysis was almost the same as the FACS analysis.

FACS from 2 retinas of 2 Thy1-CFP mice enabled us to sort 1,494–3,550 CFP-positive cells (see method and Fig. S1, gate RGCs) in 30–50 min, which was considered sufficiently fast to collect fresh cells to analyze mRNA expression. Re-analysis of the sorted cells showed that 88.9–93.4% of the cells were CFP-positive, which indicates that the FACS sorting effectively collected and concentrated the CFP-positive retinal cells (Fig. 2c). Immunostaining of the sorted cells showed that almost all the sorted cells were Brn3a- and Pax6-positive and were retinal ganglion cells (Fig. 2d and Fig. S2). To confirm that the collected cells were indeed retinal ganglion cells, their mRNAs were visualised with the Integrated Genome Browser^35^. We confirmed expected mRNA expression profiles: high expression of synaptosomal-associated protein (*Snap25*)^36^, and tubulin, beta 3 class III (*Tubb3*), which are expressed in neuronal cells and RNA binding protein with multiple splicing (*Rbpms*)^37^, which is expressed in retinal ganglion cells and low expression of *rhodopsin* (*Rho*), which is expressed in rod photoreceptors. These results further validated our FACS sorting protocol (Fig. 2e, gate RGCs in Fig. S1). Consistent with the above, CFP-negative cells collected by gate PR in Fig. S1, expressed high *Rho* levels without *Snap25, Tubb3*, and *Rbpms* expression, indicating that they contained rod photoreceptors and not ganglion cells (Fig. 2e, gate PR). From these data, we assumed the CFP-positive cells sorted by FACS, successfully enriched retinal ganglion cells and were suitable for the next experiments.

### mRNAs related to gene expression and energy metabolism were upregulated in KUS-treated retinal ganglion cells

**Figure 3:**
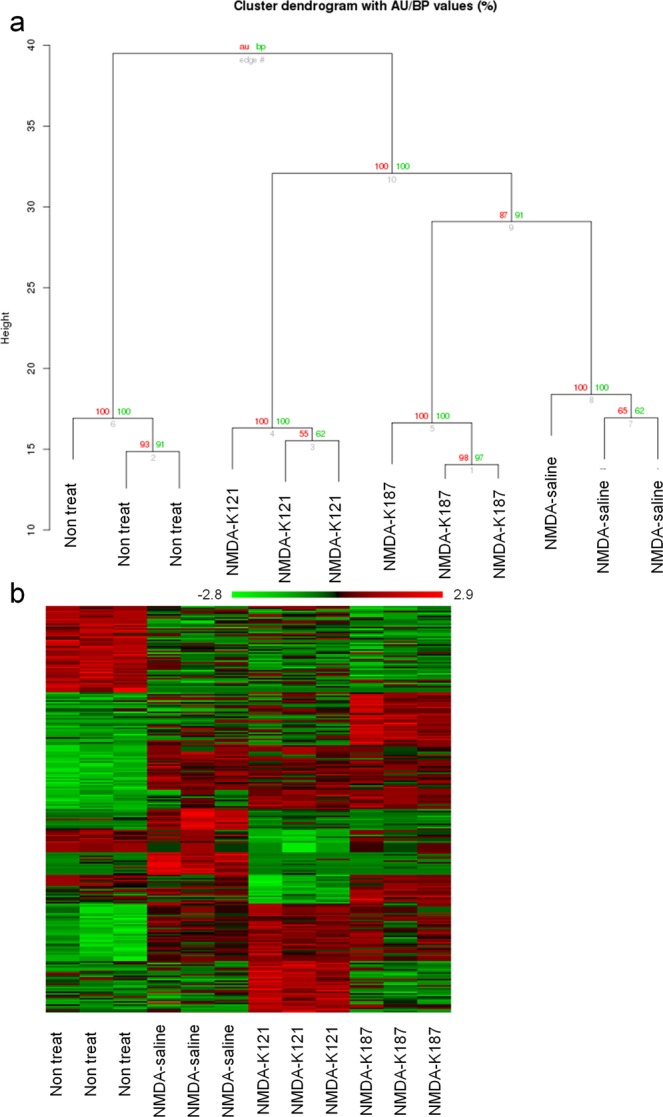
mRNA expression in retinal ganglion cells in an acute injury model. (**a**) Tree diagram of hierarchically clustered conditions. Red and green numbers show approximately unbiased p-values (AU) and bootstrap probability value (BP). (**b**) Heat map of mRNA expression of clustered conditions. Non treat: retinal ganglion cells of non-treated mice, NMDA-K121: retinal ganglion cells of NMDA-injected mice administered KUS121, NMDA-K187: retinal ganglion cells of NMDA-injected mice administered KUS187, NMDA-saline: retinal ganglion cells of NMDA-injected mice administered vehicle.

Using analysis of variance (ANOVA), 255 genes showed significant (P < 0.01) expression changes among four conditions; non-treatment (non-treat), vehicle with intravitreous injection of NMDA (NMDA-saline), KUS121 treatment with intravitreous injection of NMDA (KUS121), and KUS187 treatment with intravitreous injection of NMDA (KUS187) (Supplementary Table S1). Hierarchical clustering analyses of the 255 genes distributed samples between the conditions (Fig. 3a,b). Hierarchical clustering analyses of the ANOVA-passed genes revealed that ANOVA successfully selected genes that distinguish each condition (Fig. 3a). Moreover, the samples of the three experimental repeats showed similar patterns of upregulated and downregulated genes on heatmap (Fig. 3b). These results showed that the experimental repeats displayed great reproducibility of the gene expression profiles within each condition. While some genes showed similar expression patterns between the NMDA-saline and KUSs-treated groups, other genes showed clearly differential expression patterns (Fig. 3b). KUSs-treated groups were clearly separated from saline-treated groups. These data indicated the KUSs-treated groups have characteristic gene expression profiles distinct from the non-treat or NMDA-saline groups.

Gene ontology (GO) analysis^38^ was performed to annotate genes into biological ontology. After X-means clustering, genes in hyper cluster A and B were analyzed (Supplementary Fig. S3). Genes in hyper cluster A were found to be associated with 31 GO terms, which included RNA metabolic processes, biosynthetic processes, gene expression and metabolic processes (Table 1, Supplementary Table S2) while genes in hyper cluster B were not associated with enrichment of any GO terms.

**Table 1:** Top 15 gene ontology (GO) terms for genes whose upregulation by NMDA was attenuated in KUS-treated retinal ganglion cells.

ID	p value	Over-representation	Description	Genes
GO:0051252	0.000903	3.1	regulation of RNA metabolic process	*Camta1,Cir1,Csrnp2,Fos,Fosb,Gatad2a,Nr4a1,Rfc1*
GO:0010556	0.001284	2.9	regulation of macromolecule biosynthetic process	*Camta1,Cir1,Csrnp2,Fos,Fosb,Gatad2a,Nr4a1,Rfc1*
GO:0019219	0.001308	2.9	regulation of nucleobase-containing compound metabolic process	*Camta1,Cir1,Csrnp2,Fos,Fosb,Gatad2a,Nr4a1,Rfc1*
GO:0051171	0.001308	2.9	regulation of nitrogen compound metabolic process	*Camta1,Cir1,Csrnp2,Fos,Fosb,Gatad2a,Nr4a1,Rfc1*
GO:0031326	0.001326	2.9	regulation of cellular biosynthetic process	*Camta1,Cir1,Csrnp2,Fos,Fosb,Gatad2a,Nr4a1,Rfc1*
GO:0009889	0.001332	2.9	regulation of biosynthetic process	*Camta1,Cir1,Csrnp2,Fos,Fosb,Gatad2a,Nr4a1,Rfc1*
GO:0010468	0.00219	2.7	regulation of gene expression	*Camta1,Cir1,Csrnp2,Fos,Fosb,Gatad2a,Nr4a1,Rfc1*
GO:0060255	0.002518	2.7	regulation of macromolecule metabolic process	*Camta1,Cir1,Csrnp2,Fos,Fosb,Gatad2a,Nr4a1,Rfc1*
GO:0080090	0.003192	2.6	regulation of primary metabolic process	*Camta1,Cir1,Csrnp2,Fos,Fosb,Gatad2a,Nr4a1,Rfc1*
GO:0031323	0.003291	2.6	regulation of cellular metabolic process	*Camta1,Cir1,Csrnp2,Fos,Fosb,Gatad2a,Nr4a1,Rfc1*
GO:0034645	0.001295	2.6	cellular macromolecule biosynthetic process	*Camta1,Cir1,Csrnp2,Eif2s3y,Extl1,Fos,Fosb,Gatad2a,Nr4a1,Rfc1*
GO:0009059	0.001314	2.6	macromolecule biosynthetic process	*Camta1,Cir1,Csrnp2,Eif2s3y,Extl1,Fos,Fosb,Gatad2a,Nr4a1,Rfc1*
GO:0019222	0.003592	2.5	regulation of metabolic process	*Camta1,Cir1,Csrnp2,Fos,Fosb,Gatad2a,Nr4a1,Rfc1*
GO:0034654	0.006007	2.3	nucleobase-containing compound biosynthetic process	*Camta1,Cir1,Csrnp2,Fos,Fosb,Gatad2a,Nr4a1,Rfc1*
GO:0019438	0.006642	2.3	aromatic compound biosynthetic process	*Camta1,Cir1,Csrnp2,Fos,Fosb,Gatad2a,Nr4a1,Rfc1*

**Figure 4:**
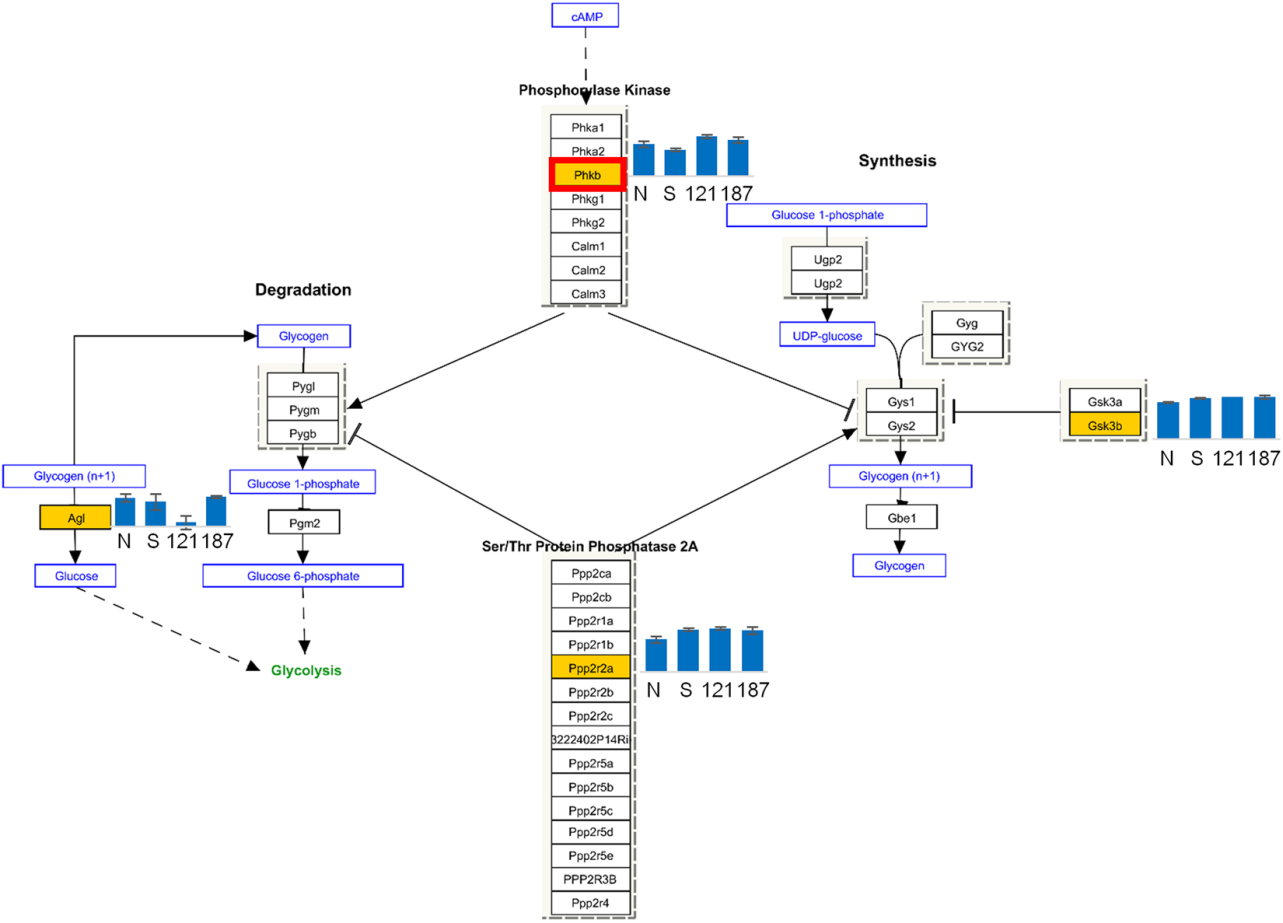
Glycogen metabolism Pathway activation in KUS-treated retinal ganglion cells. Pathway analysis of genes which were upregulated (fold change > 2) or downregulated (fold change < 0.5) between groups were done using an analysis software, Gene Spring14 (TOMY Digital Biology, http://genespring-support.com/). Pathways that were common in NMDA-saline < non-treat, KUS121 > NMDA-saline and KUS187 > NMDA-saline included glycogen metabolism pathway. Genes included in the current analysis, which passed p < 0.01 with analysis of variance (255 genes), are shown in orange color boxes. The bar graph next to the genes in the orange color boxes show the normalized expression values (N: non-treat, S: NMDA-saline, 121: KUS121, 187: KUS187). Phkb was an only gene which passed the upregulated and downregulated criteria above; downregulated in NMDA-saline compared to non-treat and was upregulated in KUS121 and KUS187 compared to NMDA-saline. The gene is shown in red frame box. non treat: retinal ganglion cells of mice without NMDA injection nor KUS treatment, NMDA-saline: retinal ganglion cells of NMDA-injected mice administered vehicle, KUS121: retinal ganglion cells of NMDA-injected mice administered KUS121, KUS187: retinal ganglion cells of NMDA-injected mice administered KUS187. In fold change analysis, >2 indicates more than 2 folds while <0.5 indicates less than 0.5 fold. For pathway analysis, A < B means statistically significant pathways of genes that were downregulated in group A by less than 0.5 fold compared to group B, and A > B means statistically significant pathways of genes that were upregulated in group A by more than 2 folds compared to group B.

Pathway analysis^39,40^ showed 4 statistically significant pathways which were common in NMDA-saline < non-treat pathways, KUS121 > NMDA-saline pathways and KUS187 > NMDA-saline pathways (Table 2). The activated pathways included the glycogen metabolism pathway (Fig. 4). There were 7 statistically significant pathways which were common in NMDA-saline > non-treat pathways, KUS121 < NMDA-saline pathways and KUS187 < NMDA-saline pathways (Table 3).

**Table 2:** Pathways which were common in NMDA-saline group < non-treat group, KUS121 group > NMDA-saline group and KUS187 group > NMDA-saline group (fold change > 2 or fold change < 0.5, respectively).

ID	Term	Matched Entities (K121 > saline)	Matched Entities (K187 > saline)	Matched Entities (saline < non-treat)	Total Entities	p value (K121 > saline)	p value (K187 > saline)	p value (saline < non-treat)
WP1251	Metapathway biotransformation	1	1	3	143	0.005723	0.004692	5.40E-09
WP317	Glycogen Metabolism	1	1	1	34	0.007623	0.006252	0.007125
WP2310	PodNet-protein-protein interactions in the podocyte	1	3	2	315	0.00952	3.61E-08	3.12E-05
WP2309	XPodNet-protein-protein interactions in the podocyte expanded by STRING	1	3	2	836	0.018951	4.31E-07	1.40E-04

saline: NMDA-saline group, K121: KUS121 group, K187: KUS187 group.

**Table 3:** Pathways which were common in NMDA-saline group > non-treat group, KUS121 group < NMDA-saline group and KUS187 group < NMDA-saline group (fold change > 2 or fold change < 0.5, respectively).

ID	Term	Matched Entities (K121 < -saline)	Matched Entities (K187 < -saline)	Matched Entities (saline > non-treat)	Total Entities	p value (K121 < saline)	p value (K187 < saline)	p value (saline > non-treat)
WP2310	PodNet-protein-protein interactions in the podocyte	3	1	1	315	1.14E-07	0.008276	0.017887
WP2309	XPodNet-protein-protein interactions in the podocyte expanded by STRING	3	2	5	836	1.36E-06	1.21E-04	1.38E-10
WP200	Complement Activation, Classical Pathway	1	1	1	17	0.002287	0.001661	0.003603
WP1560	MicroRNAs in Cardiomyocyte Hypertrophy	1	1	1	104	0.004569	0.003318	0.007193
WP29	Notch Signaling Pathway	1	1	2	47	0.004569	0.003318	1.29E-05
WP374	Prostaglandin Synthesis and Regulation	1	1	1	31	0.006846	0.004974	0.010771
WP1266	SIDS Susceptibility Pathways	1	1	2	61	0.006846	0.004974	3.85E-05

saline: NMDA-saline group, K121: KUS121 group, K187: KUS187 group.

### *Zfp*667 was upregulated in KUS121-treated retinal ganglion cells

We next performed literature search on the functions of the 255 genes whose mRNA expression changed significantly among the four groups. These genes included genes related to energy metabolism, cell proliferation, cell survival, and cell death, such as zinc finger protein 667 (*Zfp667*), phosphorylase b kinase regulatory subunit beta (*Phkb*), peroxisome proliferative activated receptor gamma coactivator 1 alpha (*Ppargc1a*), pentatricopeptide repeat domain 2 (*Ptcd2*), nucleophosmin 1 (*Npm1*), dual specificity phosphatase 18 (*Dusp18*), paternally expressed gene 10 (*Peg10*), and topoisomerase (DNA) 3 alpha (*Top3a*) (Table 4).

**Table 4:** Focused genes which were significantly changed between KUS-treated and control retinal ganglion cells.

Description	(possible) Function
*Zfp667*	may play a role in protecting cells against ischemia-reperfusion injury^41,42^
*Phkb*	plays a role in glycogen metabolism^47^
*Ppargc1a*	activate mitochondrial function^48–50^
*Ptcd2*	plays a role in post-transcriptional expression of the mitochondrial genome^51^
*Npm1*	enhances cell survival under stress conditions^52^
*Dusp18*	inactivate MAP kinases^47^
*Peg10*	promote growth and suppress apoptosis^53^
*Top3a*	adjust the DNA topological states^47^

**Figure 5:**
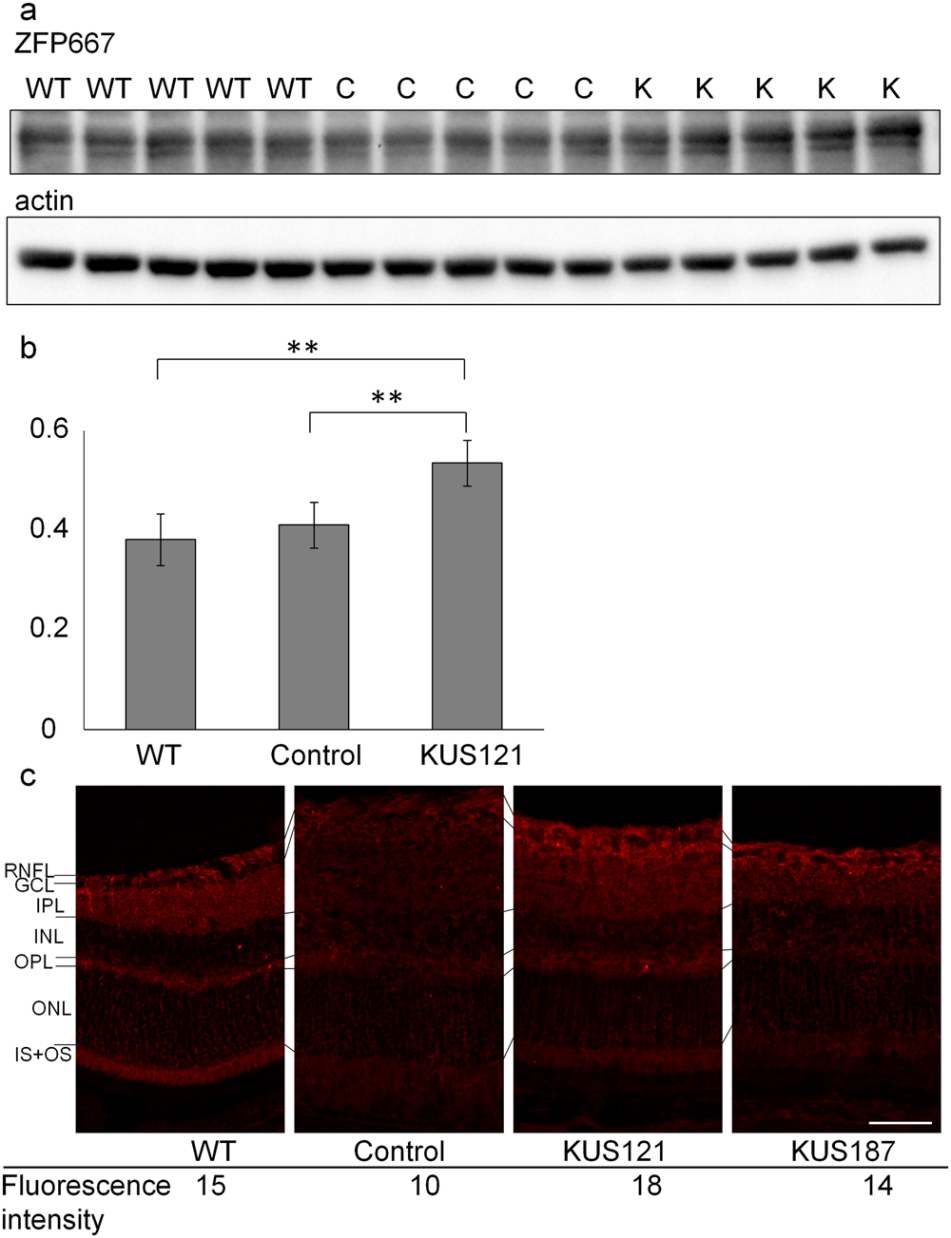
Increased expression of Zfp667 in KUS121-treated retinal ganglion cells. (**a**) Western blot analysis of NMDA-injected mice retinas with KUS121 (labelled as “K”) or vehicle (NMDA-saline as control, labelled as “C”) administration with a zing finger protein (ZFP667) antibody. Actin was used as a loading control. WT: wild-type mouse retina. Complete scans of western blots are shown in Supplementary Fig. S4. (**b**) Comparison of ZFP667 expression shown as ratio of actin. **P < 0.01, Turkey HSD. (**c**) Vertical retinal sections of mouse retina were stained with anti-ZFP667 antibody (red). Fluorescence intensity of the complex of RNFL, GCL and IPL was measured. Control: NMDA-injected mouse retina with saline administration, KUS121: NMDA-injected mouse retina administered KUS121, KUS187: NMDA-injected mouse retina administered KUS187, WT: wild-type mouse retina without NMDA injection or KUSs administration. RNFL: retinal nerve fiber layer; GCL: ganglion cell layer; IPL: inner plexiform layer; INL: inner nuclear layer; OPL: outer plexiform layer; ONL: outer nuclear layer; IS: inner segment of photoreceptors, and OS: outer segment of photoreceptors. Scale bar: 50 µm.

Of the genes studied, we focused on *Zfp667*, which has been reported to suppress apoptosis-related genes and consequently prevent cell death in ischemia-reperfusion injury^41,42^. Western blot analysis of mouse retinal proteins showed that expression levels of Zfp667 was not significantly different between non-treated retinas and saline-treated NMDA-injected retinas (NMDA-saline as control) of wild-type mice (P = 0.61, Turkey HSD, Fig. 5a,b). In contrast, expression of Zfp667 was significantly increased in KUS121-treated NMDA-injected retinas compared to the NMDA-saline group (P = 0.004, Turkey HSD, Fig. 5a,b). Immunohistochemical analysis with an anti-Zfp667 antibody showed higher expression of Zfp667 predominantly at the retinal ganglion cell layer in KUSs-treated NMDA-injected retinas compared to NMDA-saline injected retinas (Fig. 5c).

## Discussion

In this study, we successfully isolated retinal ganglion cells to a high level of purity using FACS and found that KUSs affect the expression of a wide variety of genes. These include genes involved in the regulation of energy metabolism and suppression of apoptosis in the retinal ganglion cells of an NMDA-induced retinal injury model mouse.

There have been studies in which neural cells including retinal ganglion cells were isolated using FACS with retrograde labelling^43,44^ or with antibodies^45^. In the current study, we used Thy1-CFP transgenic mice^29,30^, in which CFP is expressed in retinal ganglion cells, to omit the need for antibody reaction or the retrograde labelling step. It enabled us to collect the cells simply and quickly, which were suitable for the analysis of mRNA profiles.

In the preliminary experiments to decide the timing for evaluating gene expression after intravitreous NMDA injection, neural retina of wild-type mice was used. While intravitreous NMDA injection has been reported to damage RGCs^19^, whole retinas of mice that received intravitreous NMDA injection were used for mRNA evaluation by qRT-PCR^46^. Hence, we used neural retina in the preliminary experiments. Moreover, among the 18 genes evaluated by qRT-PCR using neural retina, *Jun* and *Fos* were included in the 255 genes evaluated by next-generation sequencing using sorted RGCs that showed significant changes in expression among the four conditions. The expression of *Jun* and *Fos* genes was upregulated by 1.63 and 3.59 times, respectively, in NMDA treated mice RGCs compared to non-treated controls.

Our experiments showed that KUS121, which has already been used in a clinical trial for ischemic retinal disease (UMIN000023979), upregulated gene expression and translation of *Zfp667* in the retinal ganglion cells. *Zfp667* has been reported to suppress apoptosis-related genes in ischemia-reperfusion injury^41,42^. KUS121 also upregulated the expression of a variety of genes such as *Phkb*, which is involved in glycogen metabolism^47^; *Ppargc1a*, which is a strong activator of mitochondrial function and a regulator of energy metabolism^48–50^; *Ptcd2*, which is involved in mitochondrial gene expression^51^; *Npm1*, which promotes cell survival under stress^52^; *Dusp18*, which may dephosphorylate and inactivate mitogen-activated protein kinase (MAPK)^47^; *Peg10*, which is anti-apoptotic^53^. On the other hand, genes that were downregulated by KUS121 included *Top3a*, which adjusts the DNA topological states during transcription^47^. Whether the translation of these genes is also altered remains to be clarified. In addition to reducing ATP consumption, these results revealed the possibility that KUS121 prevents retinal ganglion cell death through several mechanisms, including activating energy production and suppression of apoptosis. These mechanisms could be related to the modulation of VCP function or alternatively, KUSs may have additional targets, which could be involved in transcriptional control of cell survival. These possibilities need to be elucidated by further studies.

In conclusion, KUS121 can modulate gene expression profiles in retinal ganglion cells in mice, via mechanisms not yet fully elucidated, which are likely to contribute to protecting the retinal ganglion cells from NMDA-induced neurotoxicity. This study further strengthens the suitability of KUS121 as a therapeutic drug in rescuing retinal ganglion cells in eye diseases that are currently incurable, such as glaucoma.

## Methods

### Experimental animals

This study was conducted in accordance with the Association for Research in Vision and Ophthalmology (ARVO) Statement for the Use of Animals in Ophthalmic and Vision Research. All protocols were approved by the Institutional Review Board of Kyoto University Graduate School of Medicine (MedKyo 12245, 13221, 14213, 15531, 16501). B6.Cg-Tg(Thy 1-CFP) 23Jrs/J mice were obtained from the Jackson Laboratory (Bar Harbor, ME, USA) and wild-type mice (C57/BL6), which share the genetic background of Thy1-CFP mice, were purchased from Japan SLC, Inc. Mice were kept in a 14 h light/10 h dark cycle and fed *ad libitum*. Male mice aged 2 to 3 months were used for the experiments. Before intravitreous NMDA injection (5 nmol), mice were anesthetised with intraperitoneal pentobarbital (50 mg/kg) injection and pupils were dilated with tropicamide and phenylephrine eye drops (0.5% each).

### Quantitative RT-PCR of neural retinae

Changes in mRNA expression in the neural retina were examined at several time points after intravitreous NMDA injection. NMDA was intravitreally injected into wild-type mice to induce acute damage of retinal ganglion cells^17,19^. Eyeballs were enucleated 2, 4, and 6 h after NMDA injection after pentobarbital overdose. Enucleated eyeballs were immersed in cold Hanks’ balanced salt solution immediately after enucleation. Incisions were made using pinholes in the corneas, then using the incisions, the sclera was peeled to remove the mixture of the retinal pigment epithelium, choroid and sclera from the neural retina as previously described^16^. The lens and iris were removed. RNA was isolated from the neural retina using the RNeasy Mini Kit (QIAGEN, Venlo, Netherlands). The mRNA was reverse transcribed with the M-MLV reverse transcriptase (Promega, WI, USA) and then complementary DNA was amplified by PCR with SYBR premix Ex Taq polymerase (Takara Bio Inc., Shiga, Japan) and 60 °C as the annealing temperature on the 7300 Real-Time PCR System (Applied Biosystems, CA, USA). Eyes were enucleated before and 2, 4 and 6 h after NMDA injection and each eye was analysed separately.

The levels of the following mRNAs were analysed by qRT-PCR: *Nfkbia*, *Il6*, *Rela*, *Casp3*, *Fos*, *Mapk1*, *Mapk3*, *Mapk10*, *Jun*, *Tnf*, *Akt1*, *Akt2*, *Fis1*, *Mfn1*, *Mfn2*, *Dnm1l*, *Opa1*, *Hmgb1* (primers used are shown in Supplementary Table S3). Actin was used as the internal standard.

### Administration of KUSs and preparation of cell suspension for flow cytometry

Daily KUS121, KUS187 (50 mg/kg/day each), or vehicle (5% Cremophor EL (Sigma) in phosphate buffered saline (PBS)) as a control were given orally to Thy1-CFP mice using a feeding tube. Seven days after the start of the medication, NMDA (5 nmol) was intravitreally injected with a 33-gage needle^54^. Our experiment involved one-week pre-treatment with KUSs because we previously showed that it protects against NMDA injury^17^. Four hours after the NMDA injection, the retinas were collected as described in the qRT-PCR section and incubated in 0.2% papain solution (two retinas of two mice each) including glucose (1 mg/mL), DNase 1 (Worthington, 100 U/mL), superoxide dismutase (Worthington, 5 µg/mL) and catalase (Sigma, 5 µg/mL) at 8 °C for 30 min and at 28 °C for 9 min. The solution was centrifuged at 100 G for 5 min at 4 °C, cells were resuspended in a solution containing antipain (Roche, 50 µg/mL), and then centrifuged again at 100G for 5 min at 4 °C^55^. The cell pellet was resuspended in 500 µL of ice-cold Ames’ medium (with L-glutamine, without sodium bicarbonate, Sigma).

### Collection of retinal ganglion cells by flow cytometry

The cells in suspended Ames’ medium described above were sorted by FACS Aria 2 (BD Biosciences) based on the size and intensity of CFP fluorescence. Immediately after 7-Amino-Actinomycin D (BD Biosciences, Cell Viability Solution, 20 µL) were added, sorting was performed using a 85-micron nozzle into Ames’ medium at 4 °C. Forward scatter (FSC) and sideward scatter (SSC) were used to segregate retinal ganglion cells. CFP-positive cells were contained in the high FSC sub-population (area 2 in Supplementary Fig. S1a). To collect retinal ganglion cells with high purity, only cells included in both gate 4 (Supplementary Fig. S1b), which is a smaller area than area 2, and gate 5 (Supplementary Fig. S1c), which was narrower than the area 1 in Fig. 2a–c, were collected (gate RGCs). For comparison, cells included in both gate 6 (Supplementary Fig. S1b) and gate 7 (Supplementary Fig. S1c), which were considered to be photoreceptors, were collected in the same way (gate PR).

### cDNA synthesis, amplification and next-generation sequencing

The sorted cells (1,494–7,848 cells for each sample) were centrifuged at 500G for 5 min at 4 °C, suspended in 100 µL of buffer B (Prelude Direct Lysis Module, NuGEN), centrifuged again at 500G for 5 min at 4 °C, and the pellet was resuspended in 1 µL of buffer A (Prelude Direct Lysis Module, NuGEN). The lysates were then taken forward for cDNA synthesis and amplification using Ovation RNA-Seq System V2 (NuGEN) according to the manufacturer’s instructions. The amplified cDNA was purified using MinElute Reaction Cleanup Kit (QIAGEN, Venlo, Netherlands). The quality of the amplified cDNAs was analyzed by a 2100 Bioanalyzer (Agilent) and was high enough for sequencing. The concentration of cDNA as measured by Qubit (Invitrogen) was sufficient (84.7–185.0 ng/µL). The amplified cDNA was then sequenced with a next-generation sequencer (Illumina HiSeq). The experiments were repeated three times.

### Analysis of the RNA-sequencing results

The results of RNA-sequencing were mapped on reference sequence (mouse mm10, USCS genome browser) using TopHat2. Reads which formed reasonable pairs (on the same chromosome, two directionally, and distance between pair reads < 500 k b.p.) were used to calculate expression levels (61.1 ± 8.5% of reads). Reads Per Kilobase of exon model per 10 Million mapped reads (RPK10M) were calculated for terminal exons with the in-house scripts as the expression values for genes, and transferred into log_2_ scale. Genes whose maximum expression values were more than 3 among the conditions were considered as expressed genes and used for the following analyses. Then the expression value was normalized using quantile normalization methods^56^. Using analysis of variance (ANOVA), 255 genes (including isoforms) showed significant changes in expression among the four conditions (non-treat, NMDA-saline, KUS121 and KUS187, p < 0.01). Hierarchical clustering of the 255 genes that passed the ANOVA test with approximately unbiased p-values and bootstrap probability value of mRNA expression was performed with pvclust package in the statistical environment R using Euclidean distances. A heatmap was drawn with Z-value transferred expression values. Expression profiles of ANOVA-passed genes were transferred to Z-scores and clustered with the x-means method. X-means clustering was performed on the statistical environment R. The genes were divided into 12 clusters that were further categorized according to expression changes across conditions. The upregulation and downregulation of gene expression were defined as the difference in the cluster centers between conditions with more than 0.2 and less than −0.2 z-values, respectively (Supplementary Fig. S3). The clusters with upregulated expression in NMDA-saline compared to non-treat and downregulated expression in KUS121 and KUS187 compared to NMDA-saline were defined as hyper cluster A. The clusters whose expression was upregulated in NMDA-saline compared to non-treat and whose expression was upregulated in KUS121 and KUS187 compared to NMDA-saline, were defined as hyper cluster B. GO analysis of the genes included in hyper cluster A (55 genes) and B (12 genes) was performed based on hypergeometric distribution.

Pathway analysis was performed by Gene Spring14 (TOMY Digital Biology) using Wiki Pathways (http://www.wikipathways.org/index.php/WikiPathways). Genes that were upregulated (115 genes) or downregulated (57 genes) in the NMDA-saline group compared to the non-treat group, and those that were upregulated (61 genes) or downregulated (73 genes) in the KUS121 group compared to the NMDA-saline group as well as upregulated (50 genes) or downregulated (53 genes) in the KUS187 group compared to the NMDA-saline group (fold change > 2 or fold change < 0.5, respectively) were analyzed. The level of statistical significance was set to P < 0.05.

In fold change analysis, >2 indicates more than 2 folds while <0.5 indicates less than 0.5 fold. For pathway analysis, A < B means statistically significant pathways of genes that were downregulated in group A by less than 0.5 fold compared to group B, and A > B means statistically significant pathways of genes that were upregulated in group A by more than 2 folds compared to group B.

### Immunocytological evaluation of cells

Dissociated retinal cells or FACS sorted cells were fixed by adding an equal amount of 4% paraformaldehyde, centrifuged at 3000 rpm for 15 min at 4 °C. After the extra supernatant was removed, the cell suspension (200 µL) was centrifuged at 1000 rpm for 10 min using Cytospin (Thermo Scientific) to be pasted onto slides. The cells were stained with anti-Brn3a (CHEMICON) and anti-Pax6 (COVANCE) antibodies and imaged under an optical microscope (Axio Imager.A1, Zeiss).

### Immunohistological evaluation of retinas

Non-treated eyeballs or NMDA-injected eyeballs of mice treated with KUS121, KUS187 or vehicle (saline) were enucleated after pentobarbital overdose. A marking dye (Davidson) was placed on the edge of the superior conjunctiva to identify the superior portion of the retina as previously described^57^. The eyes were fixed in 4% paraformaldehyde for 24 h at 4 °C, embedded in O.C.T. compound (Sakura Finetek Japan) and frozen. Serial 16 µm O.C.T-embedded sections were cut through the dye and at the point of insertion of the optic nerve. Sections that included the center of the optic nerve head were stained with an anti-ZFP667 antibody (GeneTex) and imaged under an optical microscope (BZ-9000, Keyence) at a distance of 400 µm from the edge of the optic nerve head. The fluorescence intensity of each eye was measured in 40 µm × 300 µm squares including the retinal nerve fiber layer, the ganglion cell layer, and the inner plexiform layer using BZ II Analyzer software (Keyence).

### Western blotting of neural retinas

Neural retinas were prepared as described in the qRT-PCR section. Neural retinas and wild-type mouse brain, which was used as a positive control, were analysed with an anti-ZFP667 antibody (GeneTex). Actin was used as a loading control. The ratio of ZFP667 to actin was compared between the KUS121-treated and the control retina using an unpaired t-test.

## Supplementary information

Supplementary information.

## Data Availability

All data generated or analyzed during this study are included in this published article and its Supplementary Information files.

## References

[1] Sommer A (1991). Racial differences in the cause-specific prevalence of blindness in east Baltimore. N. Engl. J. Med..

[2] Klaver CC, Wolfs RC, Vingerling JR, Hofman A, de Jong PT (1998). Age-specific prevalence and causes of blindness and visual impairment in an older population: the Rotterdam Study. Arch. Ophthalmol..

[3] Bourne RR (2013). Causes of vision loss worldwide, 1990-2010: a systematic analysis. Lancet Glob. Health..

[4] Klein R, Klein BE (2013). The prevalence of age-related eye diseases and visual impairment in aging: current estimates. Invest. Ophthalmol. Vis. Sci..

[5] Quigley HA, Addicks EM, Green WR (1982). Optic nerve damage in human glaucoma. III. Quantitative correlation of nerve fiber loss and visual field defect in glaucoma, ischemic neuropathy, papilledema, and toxic neuropathy. Arch. Ophthalmol..

[6] Quigley HA, Dunkelberger GR, Green WR (1989). Retinal ganglion cell atrophy correlated with automated perimetry in human eyes with glaucoma. Am. J. Ophthalmol..

[7] Leske MC, Heijl A, Hyman L, Bengtsson B, Komaroff E (2004). Factors for progression and glaucoma treatment: the Early Manifest Glaucoma Trial. Curr. Opin. Ophthalmol..

[8] Vass C (2007). Medical interventions for primary open angle glaucoma and ocular hypertension. Cochrane Database Syst. Rev. Cd003167.

[9] Collaborative Normal-Tension Glaucoma Study Group (1998). The effectiveness of intraocular pressure reduction in the treatment of normal-tension glaucoma. Am. J. Ophthalmol..

[10] Ernest PJ (2013). An evidence-based review of prognostic factors for glaucomatous visual field progression. Ophthalmology..

[11] Flammer J (1994). The vascular concept of glaucoma. Surv. Ophthalmol..

[12] Marcus MW, de Vries MM, Junoy Montolio FG, Jansonius NM (2011). Myopia as a risk factor for open-angle glaucoma: a systematic review and meta-analysis. Ophthalmology..

[13] Mitchell P, Hourihan F, Sandbach J, Wang JJ (1999). The relationship between glaucoma and myopia: the Blue Mountains Eye Study. Ophthalmology..

[14] Lee JY, Sung KR, Han S, Na JH (2015). Effect of myopia on the progression of primary open-angle glaucoma. Invest. Ophthalmol. Vis. Sci..

[15] Ikeda HO (2014). Novel VCP modulators mitigate major pathologies of rd10, a mouse model of retinitis pigmentosa. Sci. Rep..

[16] Hasegawa T (2016). Neuoroprotective efficacies by KUS121, a VCP modulator, on animal models of retinal degeneration. Sci. Rep..

[17] Nakano N (2016). Neuroprotective effects of VCP modulators in mouse models of glaucoma. Heliyon..

[18] Hata M (2017). KUS121, a VCP modulator, attenuates ischemic retinal cell death via suppressing endoplasmic reticulum stress. Sci. Rep..

[19] Siliprandi R (1992). N-methyl-D-aspartate-induced neurotoxicity in the adult rat retina. Vis. Neurosci..

[20] Jeon CJ, Strettoi E, Masland RH (1998). The major cell populations of the mouse retina. J. Neurosci..

[21] Shoge K (1999). Rat retinal ganglion cells culture enriched with the magnetic cell sorter. Neurosci. Lett..

[22] Tezel G, Wax MB (2000). Increased production of tumor necrosis factor-alpha by glial cells exposed to simulated ischemia or elevated hydrostatic pressure induces apoptosis in cocultured retinal ganglion cells. J. Neurosci..

[23] Mukai S (2002). Existence of ionotropic glutamate receptor subtypes in cultured rat retinal ganglion cells obtained by the magnetic cell sorter method and inhibitory effects of 20-hydroxyecdysone, a neurosteroid, on the glutamate response. Jpn. J. Pharmacol..

[24] Huang X, Wu DY, Chen G, Manji H, Chen DF (2003). Support of retinal ganglion cell survival and axon regeneration by lithium through a Bcl-2-dependent mechanism. Invest. Ophthalmol. Vis. Sci..

[25] Hong S, Iizuka Y, Kim CY, Seong GJ (2012). Isolation of primary mouse retinal ganglion cells using immunopanning-magnetic separation. Mol. Vis..

[26] Barres BA, Silverstein BE, Corey DP, Chun LL (1988). Immunological, morphological, and electrophysiological variation among retinal ganglion cells purified by panning. Neuron..

[27] Lindsey JD, Weinreb RN (1994). Survival and differentiation of purified retinal ganglion cells in a chemically defined microenvironment. Invest. Ophthalmol. Vis. Sci..

[28] Harada C (2006). Role of apoptosis signal-regulating kinase 1 in stress-induced neural cell apoptosis *in vivo*. Am. J. Pathol..

[29] Feng G (2000). Imaging neuronal subsets in transgenic mice expressing multiple spectral variants of GFP. Neuron..

[30] Murata H (2008). Imaging mouse retinal ganglion cells and their loss *in vivo* by a fundus camera in the normal and ischemia-reperfusion model. Invest. Ophthalmol. Vis. Sci..

[31] Vidal M, Morris R, Grosveld F, Spanopoulou E (1990). Tissue-specific control elements of the Thy-1 gene. EMBO J..

[32] Caroni P (1997). Overexpression of growth-associated proteins in the neurons of adult transgenic mice. J. Neurosci. Methods..

[33] Mardis ER (2017). DNA sequencing technologies: 2006-2016. Nat. Protoc..

[34] Healy S, Khan P, Davie JR (2013). Immediate early response genes and cell transformation. Pharmacol. Ther..

[35] Nicol JW, Helt GA, Blanchard SG, Raja A, Loraine AE (2009). The Integrated Genome Browser: free software for distribution and exploration of genome-scale datasets. Bioinformatics..

[36] Oyler GA (1989). The identification of a novel synaptosomal-associated protein, SNAP-25, differentially expressed by neuronal subpopulations. J. Cell Biol..

[37] Rodriguez AR, de Sevilla Muller LP, Brecha NC (2014). The RNA binding protein RBPMS is a selective marker of ganglion cells in the mammalian retina. J. Comp. Neurol..

[38] Ashburner M (2000). Gene ontology: tool for the unification of biology. The Gene Ontology Consortium. Nat. Genet..

[39] Kelder T (2012). WikiPathways: building research communities on biological pathways. Nucleic Acids Res..

[40] Pico AR (2008). WikiPathways: pathway editing for the people. PLoS Biol..

[41] Wang G (2010). Tissue expression and subcellular localization of Mipu1, a novel myocardial ischemia-related gene. Braz. J. Med. Biol. Res..

[42] Wang K (2013). Mipu1, a novel direct target gene, is involved in hypoxia inducible factor 1-mediated cytoprotection. PLoS One..

[43] Ivanov D, Dvoriantchikova G, Barakat DJ, Nathanson L, Shestopalov VI (2008). Differential gene expression profiling of large and small retinal ganglion cells. J. Neurosci. Methods..

[44] Choudhury S (2016). Novel Methodology for Creating Macaque Retinas with Sortable Photoreceptors and Ganglion Cells. Front. Neurosci..

[45] Morrison SJ, White PM, Zock C, Anderson DJ (1999). Prospective identification, isolation by flow cytometry, and *in vivo* self-renewal of multipotent mammalian neural crest stem cells. Cell..

[46] Wada Y (2013). PACAP attenuates NMDA-induced retinal damage in association with modulation of the microglia/macrophage status into an acquired deactivation subtype. J. Mol. Neurosci..

[47] UniProt, http://www.uniprot.org/uniprot.

[48] Puigserver P (1998). A cold-inducible coactivator of nuclear receptors linked to adaptive thermogenesis. Cell..

[49] Liang H, Ward WF (2006). PGC-1alpha: a key regulator of energy metabolism. Adv. Physiol. Educ..

[50] Handschin C, Spiegelman BM (2006). Peroxisome proliferator-activated receptor gamma coactivator 1 coactivators, energy homeostasis, and metabolism. Endocr. Rev..

[51] Xu F (2008). Disruption of a mitochondrial RNA-binding protein gene results in decreased cytochrome b expression and a marked reduction in ubiquinol-cytochrome c reductase activity in mouse heart mitochondria. Biochem. J..

[52] Li J (2006). Nucleophosmin regulates cell cycle progression and stress response in hematopoietic stem/progenitor cells. J. Biol. Chem..

[53] Liu Y (2009). Suppressive effects of genomic imprinted gene PEG10 on hydrogen peroxide-induced apoptosis in L02 cells. J. Huazhong Univ. Sci. Technol. Med. Sci..

[54] Nakano N (2011). Longitudinal and simultaneous imaging of retinal ganglion cells and inner retinal layers in a mouse model of glaucoma induced by N-methyl-D-aspartate. Invest. Ophthalmol. Vis. Sci..

[55] Ma W (2013). Gene expression changes in aging retinal microglia: relationship to microglial support functions and regulation of activation. Neurobiol. Aging..

[56] Bolstad BM, Irizarry RA, Astrand M, Speed TP (2003). A comparison of normalization methods for high density oligonucleotide array data based on variance and bias. Bioinformatics..

[57] Hasegawa T (2016). Changes in morphology and visual function over time in mouse models of retinal degeneration: an SD-OCT, histology, and electroretinography study. Jpn. J. Ophthalmol..

