# Supplementary material for: Effect of VCP modulators on gene expression profiles of retinal ganglion cells in an acute injury mouse model
Source: Sci Rep. 2020 Mar 6;10:4251. doi: 10.1038/s41598-020-61160-6 (PMC7060332; doi:10.1038/s41598-020-61160-6)
Supplement: Supplementary file 1 — Supplementary information. [file 41598_2020_61160_MOESM1_ESM.pdf]

Supplementary information

Effect of VCP modulators on gene expression profiles of retinal ganglion cells in an acute injury mouse model.

Tomoko Hasegawa, Hanako Ohashi Ikeda, Norimoto Gotoh, Kei Iida, Sachiko Iwai, Noriko Nakano, Akira Kakizuka, Akitaka Tsujikawa

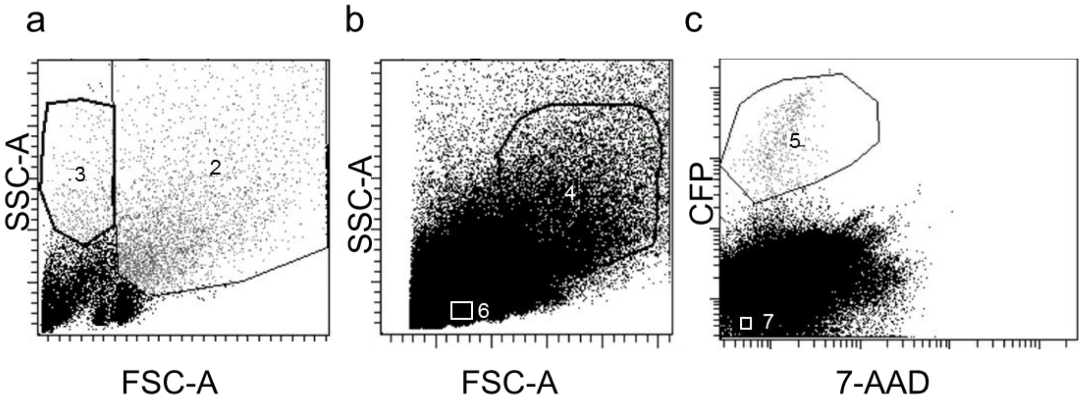

Figure S1. Analysis of retinal cells and cell sorting by flow cytometry.

(**a**) Analysis of dissociated retinal cells of Thy1-CFP mice which manifest cyan fluorescent protein (CFP) in retinal ganglion cells. The x-axis shows forward scatter (FSC)-Area and the y-axis shows side scatter (SSC)-Area. The area 2 with high FSC and SSC sub-population contained CFP-positive cells (area 1 in Fig. 2a), while no cells contained in the area 3 with low FSC subpopulation showed CFP fluorescence. (**b** and **c**) Setting of the sorting gate RGCs where retinal ganglion cells were collected. Only cells included in gate 4, which has an area smaller than area 2 in (**a**), and in gate 5, which has an area smaller than area 1 in (Fig. 2 **a**-**c**) were collected. Cells included in gates 6 and 7, which contain cells without CFP fluorescence, were collected (gate PR) for comparison.

area 2:	relatively large cells containing CFP-positive cells
area 3:	no CFP-positive cells
gate 4:	strict area of area 2
gate 5:	strict area of area 1 in Fig. 2, which contains CFP-positive cells
gate 6:	relatively small cells containing CFP-negative cells
gate 7:	containing live cells without CFP fluorescence

gate RGC:	gate 4 and gate 5 (containing CFP-positive retinal ganglion cells)
gate PR:	gates 6 and 7 (containing CFP-negative photoreceptor cells)

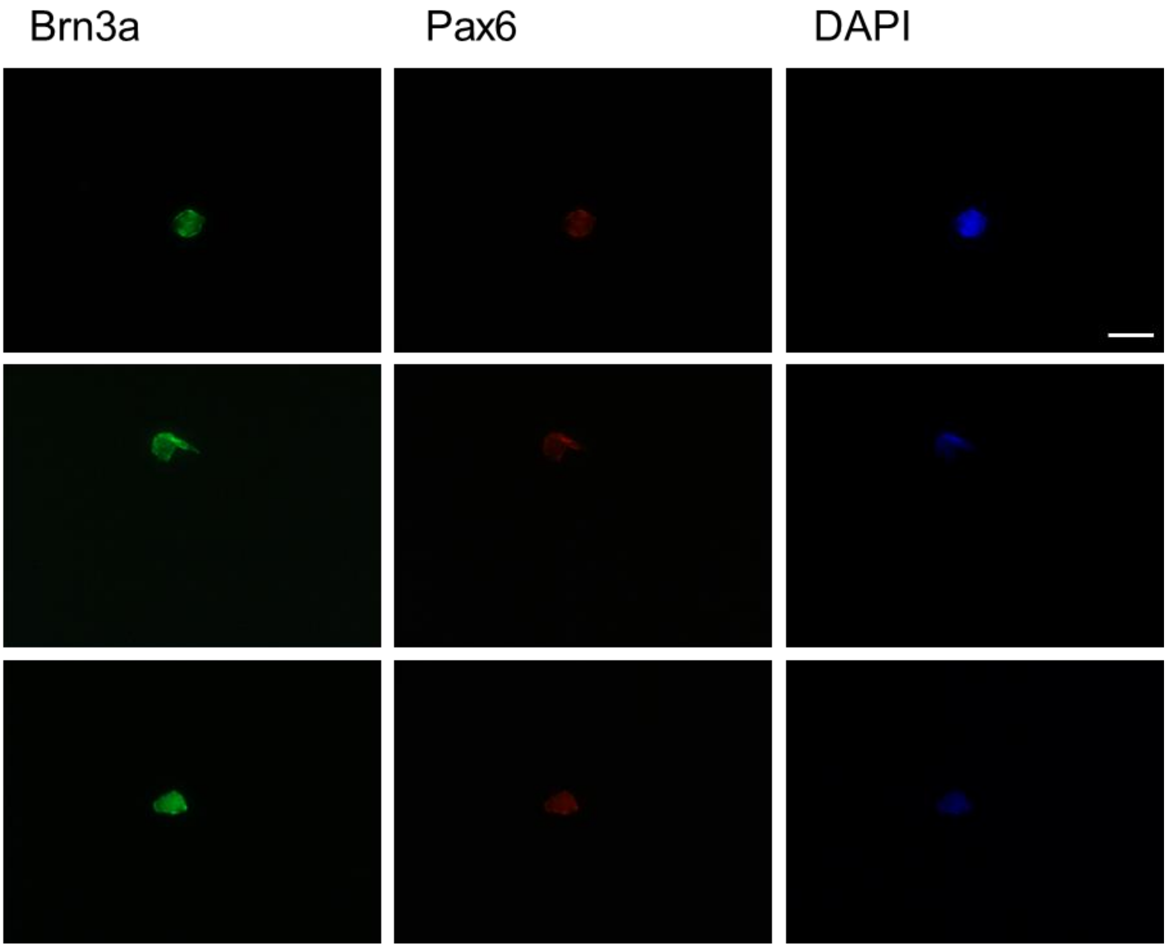

Figure S2. Immunostaining of cells sorted by flow cytometry.

The sorted cells were stained with anti-brain-specific homeobox/POU domain protein 3A (Brn3a, green) and anti-paired box genes 6 (Pax6, red) antibodies. Nuclei were counterstained with 4′,6-diamidino-2-phenylindole (DAPI, blue). Scale bar: 50 µm.

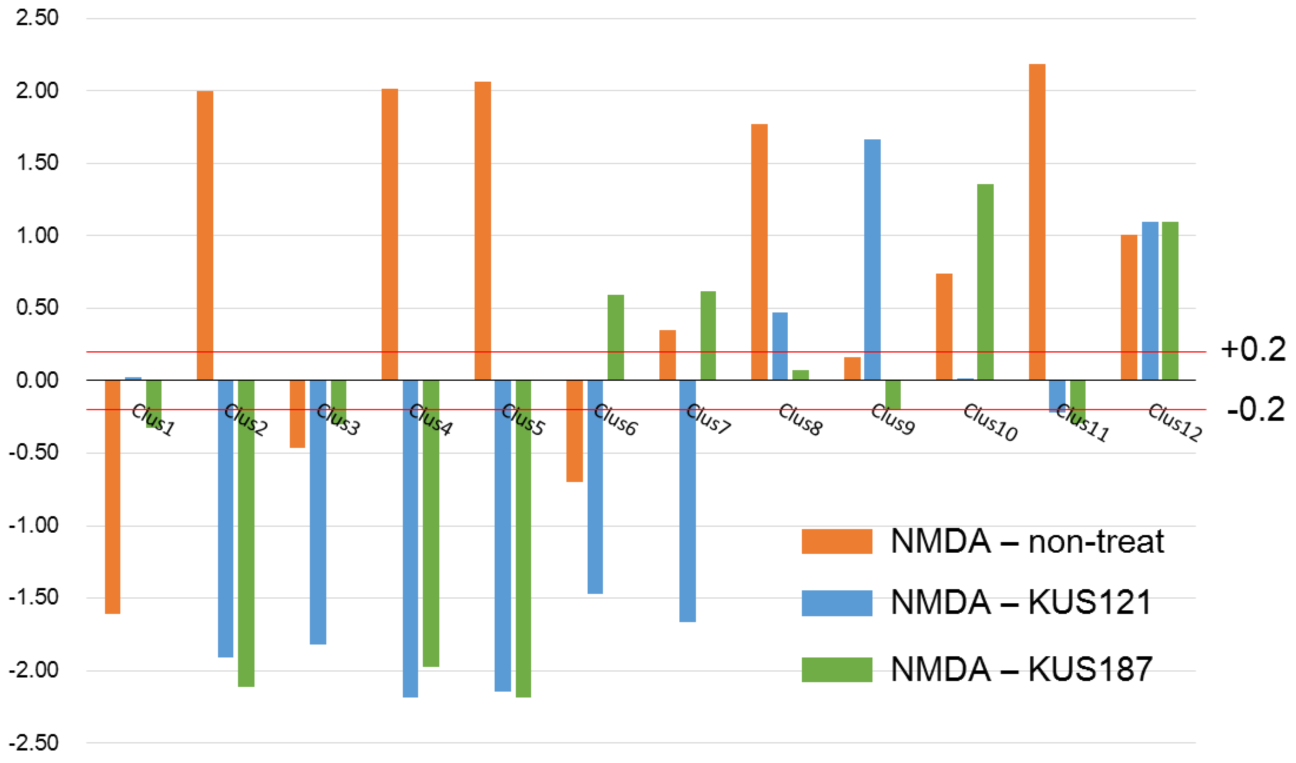

Figure S3. Differences in cluster centers among different conditions.

X-means clustering based on Z-scores classified the ANOVA-passed- genes into 12 clusters. The upregulation and downregulation of gene expression were defined as the differences in the cluster centers between conditions of more than 0.2 and less than -0.2. GO analysis was performed for hyper cluster A (including clus 2, 4, 5 and 11) and hyper cluster B (clus 12).

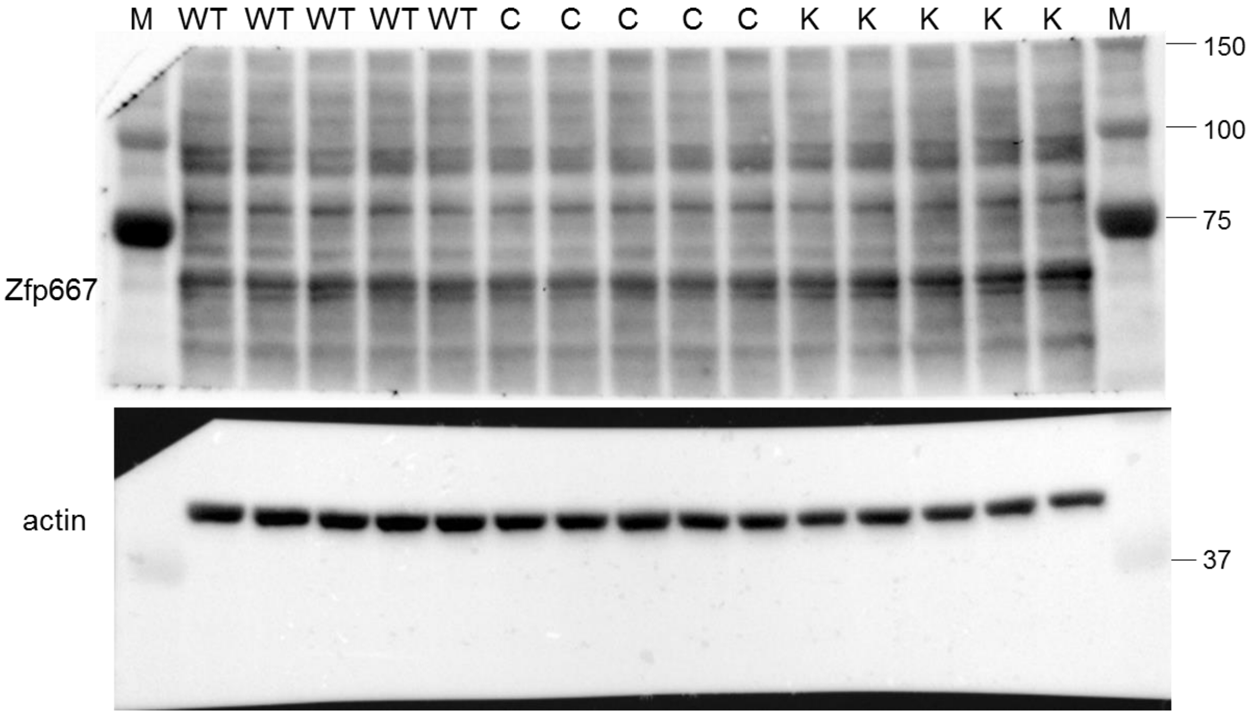

Figure S4. Complete scans of the western blots presented in the Fig. 5**a**. ZFP667 was analysed and actin was used as a loading control. M: molecular weight marker, WT: wild-type mouse retina, C: NMDA-injected mice retinas with saline administration (control), K: retinas of NMDA-injected mice administered administered KUS121.

Table S1. Genes whose mRNA expression changed significantly among four conditions.

				Relative expression			
Hyper cluster	cluster	ID	Description	Non-treat	NMDA-saline	KUS121	KUS187
-	Clus_1	NM_027049	*1700008O03Rik*	4.167	1.823	0.383	0.657
-	Clus_1	NM_008161	*Gpx3*	3.747	0.120	0.000	0.000
-	Clus_1	NM_001081493	*Cartpt*	9.513	8.580	7.697	8.540
-	Clus_1	NM_013732	*Cartpt*	9.513	8.580	7.697	8.540
-	Clus_1	NM_001099308.p2	*Gm14391*	3.640	1.853	2.693	0.623
-	Clus_1	NM_018858	*Pebp1*	8.850	8.153	7.917	7.710
-	Clus_1	NM_001163379	*Agpat1*	4.993	2.303	2.683	2.853
-	Clus_1	NM_018862	*Agpat1*	4.993	2.303	2.683	2.853
-	Clus_1	NM_021391	*Ppp1r1a*	5.790	2.507	3.877	4.817
-	Clus_1	NM_145837	*Il17d*	3.287	1.197	0.127	0.000
-	Clus_1	NR_045326	*A330009N23Rik*	3.370	0.000	0.000	0.000
-	Clus_1	NR_045327	*A330009N23Rik*	3.370	0.000	0.000	0.000
-	Clus_1	NR_045328	*A330009N23Rik*	3.370	0.000	0.000	0.000
-	Clus_1	NR_029764	*Mir331*	3.713	0.000	0.563	1.010
-	Clus_1	NM_001168577	*Nat2*	3.487	0.000	0.313	0.120
-	Clus_1	NM_010874	*Nat2*	3.487	0.000	0.313	0.120
-	Clus_1	NM_008495	*Lgals1*	5.063	2.580	1.223	1.360

-	Clus_1	NM_175106	*Tmem177*	2.793	0.067	0.643	2.230
-	Clus_1	NM_026610	*Ndufb4*	8.597	7.030	7.010	7.397
-	Clus_1	NM_013588	*Lrrc23*	4.250	0.000	0.000	1.077
-	Clus_1	NM_001170401	*Tmem229b*	4.220	3.097	3.003	2.103
-	Clus_1	NM_178745	*Tmem229b*	4.227	3.097	3.007	2.107
-	Clus_1	NM_001083945	*Rsph3b*	6.057	4.813	4.600	3.720
-	Clus_1	NM_145922	*Kcnc4*	6.423	5.027	4.940	4.230
-	Clus_1	NM_080456	*Mrps6*	7.303	5.327	5.660	4.303
-	Clus_1	NM_001168516	*Zdhhc24*	6.943	3.990	5.903	4.477
-	Clus_1	NM_001168517	*Zdhhc24*	6.943	3.990	5.903	4.477
-	Clus_1	NM_027476	*Zdhhc24*	6.943	3.990	5.903	4.477
-	Clus_1	NM_001033249	*Zfp583*	3.720	2.393	3.903	0.830
-	Clus_1	NM_023172	*Ndufb9*	8.267	7.177	7.213	7.193
-	Clus_1	NM_001033313	*Pdap1*	3.013	2.600	0.710	0.247
-	Clus_1	NM_009942	*Cox5b*	8.640	7.520	7.560	5.803
-	Clus_1	NM_198610	*Igsf21*	5.567	2.040	0.683	1.703
-	Clus_1	NM_145488	*Pex6*	4.870	3.467	4.907	1.480
-	Clus_1	NM_001025246	*Trp53i11*	3.497	1.127	3.197	0.693
-	Clus_1	NM_027215	*Tmem147*	8.193	6.167	5.040	6.217
-	Clus_1	NM_177606	*Plekhh2*	5.023	2.187	4.063	4.753
-	Clus_1	NM_022325	*Ctsz*	6.220	4.277	3.117	3.923
-	Clus_1	NM_009112	*S100a10*	8.663	7.713	8.803	6.820
-	Clus_1	NM_019561	*Ensa*	7.387	7.070	6.033	5.840

-	Clus_1	NM_013471	*Anxa4*	3.257	0.443	0.000	0.093
-	Clus_1	NM_023374	*Sdhb*	7.520	6.333	6.053	5.370
-	Clus_1	NM_026644	*Agpat4*	5.513	4.770	1.333	1.953
-	Clus_1	NM_007625	*Cbx4*	3.567	1.453	3.247	3.020
-	Clus_1	NM_018808	*Dnajb1*	7.653	6.630	6.177	5.947
-	Clus_1	NM_026784	*Pmvk*	7.167	6.540	6.327	5.597
-	Clus_1	NM_027348	*Pmvk*	7.167	6.540	6.327	5.597
-	Clus_1	NM_001145780	*Use1*	7.577	6.887	5.607	6.280
-	Clus_1	NM_025917	*Use1*	7.577	6.887	5.607	6.280
-	Clus_1	NM_011882	*Rnasel*	5.463	3.157	5.513	3.950
-	Clus_1	NM_028270	*Aldh1b1*	1.973	0.100	0.090	0.000
-	Clus_1	NM_007750	*Cox8a*	9.017	8.593	7.647	7.220
-	Clus_1	NM_013659	*Sema4b*	4.283	3.263	3.550	2.537
-	Clus_1	NM_001134717	*2810006K23Rik*	4.043	1.420	3.603	1.260
A	Clus_2	NM_024285	*Bves*	0.000	3.697	0.000	0.000
A	Clus_2	NM_010104	*Edn1*	0.350	3.317	0.307	0.000
A	Clus_2	NM_001113346	*Gatad2a*	4.413	5.743	4.827	4.290
A	Clus_2	NM_001286450	*Gatad2a*	4.413	5.743	4.827	4.290
A	Clus_2	NM_145596	*Gatad2a*	4.413	5.743	4.827	4.290
A	Clus_2	NM_001173372	*Fxyd4*	0.000	4.517	0.863	1.017
A	Clus_2	NM_033648	*Fxyd4*	0.000	4.517	0.863	1.017
A	Clus_2	NM_181409	*Mtmr11*	6.513	7.680	6.027	6.333
A	Clus_2	NM_011824	*Grem1*	1.877	2.377	0.267	0.293

A	Clus_2	NM_025653	*3110001I22Rik*	0.000	2.433	0.513	0.000
A	Clus_2	NM_001177594	*Slc8b1*	0.000	2.653	0.000	0.007
A	Clus_2	NM_001177595	*Slc8b1*	0.000	2.653	0.000	0.007
A	Clus_2	NM_133221	*Slc8b1*	0.000	2.653	0.000	0.007
-	Clus_3	NM_001256195	*Eif4g3*	8.750	8.687	7.743	8.477
-	Clus_3	NM_001256198	*Eif4g3*	8.750	8.687	7.743	8.477
-	Clus_3	NM_172703	*Eif4g3*	8.750	8.687	7.743	8.477
-	Clus_3	NM_001077631	*Vwa9*	6.083	4.807	2.670	5.500
-	Clus_3	NM_175153	*Vwa9*	6.083	4.807	2.670	5.500
-	Clus_3	NM_198326	*Nsfl1c*	7.653	7.190	5.400	6.090
-	Clus_3	NM_027801	*2610015P09Rik*	4.047	3.770	0.000	1.247
-	Clus_3	NM_145471	*Lrrc14*	4.013	4.443	0.907	3.840
-	Clus_3	NR_040257	*2210408F21Rik*	5.327	4.373	1.897	4.147
-	Clus_3	NR_040258	*2210408F21Rik*	5.327	4.373	1.897	4.147
-	Clus_3	NR_040259	*2210408F21Rik*	5.327	4.373	1.897	4.147
-	Clus_3	NR_040260	*2210408F21Rik*	5.327	4.373	1.897	4.147
-	Clus_3	NR_040261	*2210408F21Rik*	5.327	4.373	1.897	4.147
-	Clus_3	NR_040262	*2210408F21Rik*	5.327	4.373	1.897	4.147
A	Clus_4	NM_019578	*Extl1*	0.420	2.793	0.000	0.150
A	Clus_4	NM_011413	*C4a*	0.000	2.960	0.000	0.213
A	Clus_4	NM_001159650	*Crybb3*	0.000	4.063	0.000	0.883
A	Clus_4	NM_021352	*Crybb3*	0.000	4.063	0.000	0.883
A	Clus_4	NM_030728	*9930013L23Rik*	0.903	3.617	0.687	0.097

A	Clus_4	NR_030604	*Mir872*	0.927	4.603	0.000	0.723
A	Clus_4	NM_201361	*Rmdn2*	0.803	4.613	0.263	0.783
A	Clus_5	NR_037311	*Mir3473*	0.000	5.173	0.000	0.000
A	Clus_5	NR_040692	*Gm10664*	0.513	4.747	0.540	0.000
A	Clus_5	NM_001201389	*Gm11128*	0.000	2.707	0.000	0.000
A	Clus_5	NM_027017	*3300002I08Rik*	0.000	4.743	0.000	0.000
A	Clus_5	NM_009410	*Top3a*	0.823	3.727	0.000	0.323
A	Clus_5	NM_001199556	*AW551984*	0.177	2.603	0.153	0.000
A	Clus_5	NM_178737	*AW551984*	0.177	2.603	0.153	0.000
-	Clus_6	NM_001081326	*Agl*	3.477	2.977	0.477	3.573
-	Clus_6	NM_001205067	*Jkamp*	7.203	5.777	4.130	7.057
-	Clus_6	NM_024205	*Jkamp*	7.203	5.777	4.130	7.057
-	Clus_6	NM_178378	*Iqcg*	4.790	4.823	1.713	4.723
-	Clus_6	NM_001271397	*Nol8*	6.280	4.563	1.797	5.887
-	Clus_6	NR_073167	*Nol8*	6.280	4.563	1.797	5.887
-	Clus_6	NR_073168	*Nol8*	6.280	4.563	1.797	5.887
-	Clus_7	NM_031998	*Cep41*	1.437	0.087	0.127	4.333
-	Clus_7	NM_021557	*Rdh11*	4.060	4.317	0.830	5.110
-	Clus_7	NM_022813	*Scamp2*	2.693	3.950	0.090	3.797
-	Clus_7	NM_181586	*Sirt6*	2.173	2.897	0.540	4.197
-	Clus_7	NR_045463	*4930480K15Rik*	5.493	6.353	3.907	6.130
-	Clus_7	NM_001163430	*Sirt6*	2.803	3.860	0.660	4.657
-	Clus_7	NM_181407	*Me3*	2.637	1.730	0.510	4.770

-	Clus_7	NM_021280	*Plcg1*	3.277	3.387	0.273	5.597
-	Clus_7	NM_153413	*Dock3*	5.920	5.860	4.590	6.017
-	Clus_7	NM_008453	*Klf3*	1.957	3.200	0.260	2.813
-	Clus_7	NM_026430	*Uxs1*	2.967	5.643	1.943	6.053
-	Clus_8	NM_013642	*Dusp1*	3.373	8.730	8.687	8.317
-	Clus_8	NM_178892	*Tiparp*	0.783	4.153	5.320	5.847
-	Clus_8	NR_030733	*1700086L19Rik*	1.870	6.530	7.003	6.780
-	Clus_8	NR_030734	*1700086L19Rik*	1.870	6.530	7.003	6.780
-	Clus_8	NR_030735	*1700086L19Rik*	1.870	6.530	7.003	6.780
-	Clus_8	NM_010638	*Klf9*	4.003	6.053	7.007	5.660
-	Clus_8	NM_019827	*Gsk3b*	6.723	7.447	7.667	7.690
-	Clus_8	NM_019632	*Napb*	7.457	8.503	8.743	8.610
-	Clus_8	NM_011221	*Purb*	6.633	7.473	7.907	7.440
-	Clus_8	NM_025824	*Bzw1*	5.187	7.383	7.690	7.460
-	Clus_8	NM_028032	*Ppp2r2a*	5.080	6.627	6.823	6.533
-	Clus_8	NM_172289	*Slc36a4*	2.877	6.613	7.440	6.157
-	Clus_8	NM_001199242	*Kcnip4*	9.293	9.790	9.957	9.717
-	Clus_8	NM_001199243	*Kcnip4*	9.293	9.790	9.957	9.717
-	Clus_8	NM_001199244	*Kcnip4*	9.293	9.790	9.957	9.717
-	Clus_8	NM_001199245	*Kcnip4*	9.293	9.790	9.957	9.717
-	Clus_8	NM_030265	*Kcnip4*	9.293	9.790	9.957	9.717
-	Clus_8	NM_001252313	*Ncor1*	5.523	6.890	7.737	6.643
-	Clus_8	NM_011308	*Ncor1*	5.523	6.890	7.737	6.643

-	Clus_8	NM_021458	*Fzd3*	3.857	5.320	6.143	5.807
-	Clus_8	NM_030108	*Tmem33*	4.077	5.243	6.177	5.587
-	Clus_8	NM_198711	*Col25a1*	1.280	3.487	4.683	4.930
-	Clus_8	NM_001244952	*Col25a1*	1.293	3.310	4.597	4.777
-	Clus_8	NM_029838	*Col25a1*	1.293	3.310	4.597	4.777
-	Clus_8	NM_001037726	*Creb1*	3.487	4.920	5.420	5.293
-	Clus_8	NM_009952	*Creb1*	3.487	4.920	5.420	5.293
-	Clus_8	NM_133828	*Creb1*	3.487	4.920	5.420	5.293
-	Clus_8	NM_009621	*Adamts1*	0.650	4.473	4.457	4.270
-	Clus_8	NM_007913	*Egr1*	3.277	8.300	8.173	8.743
-	Clus_8	NM_027629	*Pgm2l1*	6.410	7.733	7.943	7.853
-	Clus_8	NM_007570	*Btg2*	2.580	5.020	5.153	5.827
-	Clus_8	NM_027439	*Atp6ap2*	8.923	9.680	9.853	9.370
-	Clus_8	NM_198163	*Rab35*	2.787	4.993	5.450	3.990
-	Clus_8	NM_176928	*Brwd1*	5.297	6.717	6.983	7.367
-	Clus_8	NM_001036684	*Atp2b2*	7.433	8.550	8.530	8.203
-	Clus_8	NM_009723	*Atp2b2*	7.433	8.550	8.530	8.203
-	Clus_9	NM_001033171	*Klrg2*	0.000	0.000	3.407	0.003
-	Clus_9	NR_040359	*Gm14204*	0.000	3.633	3.490	0.507
-	Clus_9	NM_203491	*Chrm2*	0.000	0.207	4.123	2.670
-	Clus_9	NR_030677	*BC025920*	1.467	0.830	4.250	0.000
-	Clus_9	NM_199446	*Phkb*	6.080	5.020	7.553	6.883
-	Clus_9	NM_008904	*Ppargc1a*	4.330	4.360	6.277	5.283

-	Clus_9	NR_027710	*Ppargc1a*	4.330	4.360	6.277	5.283
-	Clus_9	NM_001286009	*Tubgcp2*	2.827	0.243	3.417	3.280
-	Clus_9	NM_026873	*Ptcd2*	4.163	3.143	5.867	2.247
-	Clus_9	NM_023160	*Cml1*	0.893	0.660	4.650	1.310
-	Clus_9	NM_001039162	*Clip2*	0.000	1.733	3.567	1.230
-	Clus_9	NM_009990	*Clip2*	0.000	1.733	3.567	1.230
-	Clus_9	NM_001281466	*Mroh2a*	1.167	1.290	3.937	0.580
-	Clus_9	NM_173745	*Dusp18*	0.243	2.717	3.037	0.693
-	Clus_9	NM_001024928	*Zfp667*	4.420	3.953	5.907	4.427
-	Clus_9	NR_037237	*Mir3076*	1.150	0.737	3.467	0.343
-	Clus_9	NM_008217	*Has3*	1.080	1.220	2.857	1.117
-	Clus_9	NM_027088	*Bap1*	5.360	4.223	6.370	5.023
-	Clus_9	NM_011648	*Tshr*	0.230	0.090	2.933	0.127
-	Clus_9	NM_001252260	*Npm1*	8.963	9.207	10.540	7.943
-	Clus_9	NM_008722	*Npm1*	8.963	9.207	10.540	7.943
-	Clus_9	NM_020502	*Tas2r108*	0.000	1.573	3.793	0.000
-	Clus_9	NM_001083887	*Pars2*	0.940	0.170	3.377	0.013
-	Clus_9	NM_001285783	*Pars2*	0.940	0.170	3.377	0.013
-	Clus_9	NM_030711	*Erap1*	0.330	0.067	3.490	0.397
-	Clus_9	NM_172689	*Ddx58*	0.823	0.830	2.957	0.423
-	Clus_9	NM_001025576	*Ccdc141*	0.523	1.410	3.957	0.663
-	Clus_9	NM_001040611	*Peg10*	0.000	2.220	3.397	1.427
-	Clus_9	NM_130877	*Peg10*	0.000	2.220	3.397	1.427

-	Clus_9	NM_021889	*Syt9*	4.097	5.187	5.040	3.223
-	Clus_9	NM_001007581	*2810408M09Rik*	2.283	2.403	4.360	0.190
-	Clus_9	NM_001162532	*Fam174b*	2.670	1.733	4.497	0.710
-	Clus_9	NM_008140	*Gnat1*	2.450	2.907	5.113	2.420
-	Clus_10	NM_008199	*H2-Bl*	0.000	0.360	0.000	3.220
-	Clus_10	NM_001190717	*Dbf4*	0.000	0.000	0.000	3.297
-	Clus_10	NM_013726	*Dbf4*	0.000	0.000	0.000	3.297
-	Clus_10	NM_001161800	*Klhl7*	6.337	7.603	7.357	8.300
-	Clus_10	NM_026448	*Klhl7*	6.337	7.603	7.357	8.300
-	Clus_10	NM_001198571	*Abi2*	6.857	7.303	7.570	8.083
-	Clus_10	NM_198127	*Abi2*	6.857	7.303	7.570	8.083
-	Clus_10	NR_030480	*Mir698*	0.000	3.613	0.000	3.340
-	Clus_10	NM_145571	*Mob1a*	0.237	2.687	1.270	5.653
-	Clus_10	NM_001080755	*Zzz3*	3.370	5.387	3.963	5.340
-	Clus_10	NM_198416	*Zzz3*	3.370	5.387	3.963	5.340
-	Clus_10	NM_001033210	*Pls1*	0.000	2.360	0.967	3.837
-	Clus_10	NM_001081208	*Hs3st5*	5.063	3.867	5.463	5.993
-	Clus_10	NM_001253355	*Hs3st5*	5.063	3.867	5.463	5.993
-	Clus_10	NM_001253356	*Hs3st5*	5.063	3.867	5.463	5.993
-	Clus_10	NM_001033954	*Calca*	3.367	3.953	5.360	6.043
-	Clus_10	NM_022025	*Slc5a7*	0.000	1.903	3.930	5.323
-	Clus_10	NM_173403	*Slc10a4*	0.000	1.870	1.490	5.433
-	Clus_10	NM_025429	*Serpinb1a*	0.000	0.273	0.000	3.547

-	Clus_10	NM_153167	*Dcaf10*	2.697	4.233	3.953	5.243
-	Clus_10	NM_026878	*Rasl11b*	0.330	1.583	0.170	3.460
-	Clus_10	NR_027380	*Gm3219*	0.460	1.427	0.310	5.387
-	Clus_10	NM_029250	*Etnk1*	7.513	7.813	7.880	8.373
-	Clus_10	NM_009988	*Cxadr*	0.860	2.903	1.133	6.003
-	Clus_10	NM_026278	*Lrp2bp*	0.710	1.277	0.533	4.413
-	Clus_10	NM_021509	*Moxd1*	0.000	1.383	3.923	3.817
-	Clus_10	NM_001024468	*Bcat1*	5.753	5.747	6.390	6.353
-	Clus_10	NM_007532	*Bcat1*	5.753	5.747	6.390	6.353
-	Clus_10	NM_183031	*Gpr183*	0.727	0.233	0.783	2.750
-	Clus_10	NM_001283063	*Ewsr1*	5.780	7.100	6.733	7.410
-	Clus_10	NM_001197024	*Unkl*	4.583	5.573	5.167	5.837
-	Clus_10	NM_028789	*Unkl*	4.583	5.573	5.167	5.837
-	Clus_10	NM_001039195	*Gria2*	8.757	9.820	8.980	10.147
A	Clus_11	NM_008036	*Fosb*	0.273	7.617	7.697	7.560
A	Clus_11	NM_010234	*Fos*	2.657	9.537	9.583	10.067
A	Clus_11	NM_010591	*Jun*	3.887	6.320	6.640	7.023
A	Clus_11	NR_102329	*Gm4432*	0.377	5.983	4.777	5.257
A	Clus_11	NM_010479	*Hspa1a*	0.673	4.253	3.567	4.610
A	Clus_11	NM_001195565	*Camta1*	3.847	6.483	5.913	6.143
A	Clus_11	NM_009427	*Tob1*	4.470	7.247	6.427	6.867
A	Clus_11	NM_001170433	*Ppfibp1*	8.033	9.173	8.583	9.030
A	Clus_11	NM_026221	*Ppfibp1*	8.033	9.173	8.583	9.030

A	Clus_11	NM_018811	*Abhd2*	1.600	5.317	4.507	4.573
A	Clus_11	NM_001164198	*Prkacb*	7.747	8.713	8.907	8.580
A	Clus_11	NM_001164199	*Prkacb*	7.747	8.713	8.907	8.580
A	Clus_11	NM_001164200	*Prkacb*	7.747	8.713	8.907	8.580
A	Clus_11	NM_011100	*Prkacb*	7.747	8.713	8.907	8.580
A	Clus_11	NM_153407	*Csrnp2*	4.160	6.450	6.330	5.720
A	Clus_11	NM_025854	*Cir1*	1.123	5.393	4.323	4.677
A	Clus_11	NM_001127685	*BC048943*	3.210	6.143	7.037	6.463
A	Clus_11	NR_034046	*Snora61*	1.493	5.510	5.253	5.790
A	Clus_11	NM_011258	*Rfc1*	2.387	5.817	5.513	5.367
A	Clus_11	NM_001166507	*Sec14l1*	4.170	5.317	4.977	4.660
A	Clus_11	NM_138756	*Slc25a36*	5.333	6.580	6.723	6.680
A	Clus_11	NM_011817	*Gadd45g*	3.097	5.577	5.477	5.200
A	Clus_11	NM_010444	*Nr4a1*	2.560	5.573	5.300	5.063
A	Clus_11	NM_007889	*Dvl3*	2.517	4.993	4.580	3.593
A	Clus_11	NM_012011	*Eif2s3y*	6.777	8.657	8.423	8.573
A	Clus_11	NM_009170	*Shh*	2.700	5.063	4.833	5.517
A	Clus_11	NM_178619	*1810026J23Rik*	3.320	6.050	5.870	4.780
A	Clus_11	NM_001166506	*Sec14l1*	3.987	5.093	4.810	4.653
B	Clus_12	NM_153287	*Csrnp1*	0.000	1.600	3.617	2.823
B	Clus_12	NR_028573	*Snora24*	0.000	3.833	5.150	4.920
B	Clus_12	NM_009573	*Zic1*	0.247	1.710	3.297	3.970
B	Clus_12	NM_011794	*Bpnt1*	3.787	4.857	5.953	6.327

B	Clus_12	NM_198866	*Dbpht2*	4.893	6.417	7.197	7.693
B	Clus_12	NM_027603	*4921511H03Rik*	4.203	6.347	6.837	6.893
B	Clus_12	NM_001081956	*Akap17b*	3.633	4.600	5.730	5.373
B	Clus_12	NM_198103	*Exoc8*	2.270	2.783	3.613	3.280
B	Clus_12	NM_001079847	*Gpr64*	0.693	1.853	4.580	4.803
B	Clus_12	NM_001079848	*Gpr64*	0.693	1.853	4.580	4.803
B	Clus_12	NM_001079857	*Gpr64*	0.693	1.853	4.580	4.803
B	Clus_12	NM_178712	*Gpr64*	0.693	1.853	4.580	4.803

Non-treat: non-treatment,

NMDA-saline: vehicle with intravitreous injection of NMDA,

KUS121: KUS121 treatment with intravitreous injection of NMDA

KUS187: KUS187 treatment with intravitreous injection of NMDA

Clus: Cluster

Genes are in order of clusters.

Table S2. Gene ontology terms for genes in hyper cluster A, whose upregulation by NMDA was attenuated in KUS-treated retinal ganglion cells.

ID	p value	Over-representation	Description	Genes
GO:0051252	0.000903	3.1	regulation of RNA metabolic process	*Camta1,Cir1,Csrnp2,Fos, Fosb,Gatad2a,Nr4a1,Rfc1*
GO:0010556	0.001284	2.9	regulation of macromolecule biosynthetic process	*Camta1,Cir1,Csrnp2,Fos, Fosb,Gatad2a,Nr4a1,Rfc1*
GO:0019219	0.001308	2.9	regulation of nucleobase-containing compound metabolic process	*Camta1,Cir1,Csrnp2,Fos, Fosb,Gatad2a,Nr4a1,Rfc1*
GO:0051171	0.001308	2.9	regulation of nitrogen compound metabolic process	*Camta1,Cir1,Csrnp2,Fos, Fosb,Gatad2a,Nr4a1,Rfc1*
GO:0031326	0.001326	2.9	regulation of cellular biosynthetic process	*Camta1,Cir1,Csrnp2,Fos, Fosb,Gatad2a,Nr4a1,Rfc1*
GO:0009889	0.001332	2.9	regulation of biosynthetic process	*Camta1,Cir1,Csrnp2,Fos, Fosb,Gatad2a,Nr4a1,Rfc1*
GO:0010468	0.00219	2.7	regulation of gene expression	*Camta1,Cir1,Csrnp2,Fos, Fosb,Gatad2a,Nr4a1,Rfc1*
GO:0060255	0.002518	2.7	regulation of macromolecule metabolic process	*Camta1,Cir1,Csrnp2,Fos, Fosb,Gatad2a,Nr4a1,Rfc1*
GO:0080090	0.003192	2.6	regulation of primary	*Camta1,Cir1,Csrnp2,Fos,*

			metabolic process	*Fosb,Gatad2a,Nr4a1,Rfc1*
GO:0031323	0.003291	2.6	regulation of cellular metabolic process	*Camta1,Cir1,Csrnp2,Fos, Fosb,Gatad2a,Nr4a1,Rfc1*
GO:0034645	0.001295	2.6	cellular macromolecule biosynthetic process	*Camta1,Cir1,Csrnp2,Eif2s3y,Extl1,Fos,Fosb,Gatad2a,Nr4a1,Rfc1*
GO:0009059	0.001314	2.6	macromolecule biosynthetic process	*Camta1,Cir1,Csrnp2,Eif2s3y,Extl1,Fos,Fosb,Gatad2a,Nr4a1,Rfc1*
GO:0019222	0.003592	2.5	regulation of metabolic process	*Camta1,Cir1,Csrnp2,Fos, Fosb,Gatad2a,Nr4a1,Rfc1*
GO:0034654	0.006007	2.3	nucleobase-containing compound biosynthetic process	*Camta1,Cir1,Csrnp2,Fos, Fosb,Gatad2a,Nr4a1,Rfc1*
GO:0019438	0.006642	2.3	aromatic compound biosynthetic process	*Camta1,Cir1,Csrnp2,Fos, Fosb,Gatad2a,Nr4a1,Rfc1*
GO:0018130	0.006708	2.3	heterocycle biosynthetic process	*Camta1,Cir1,Csrnp2,Fos, Fosb,Gatad2a,Nr4a1,Rfc1*
GO:0044271	0.006932	2.3	cellular nitrogen compound biosynthetic process	*Camta1,Cir1,Csrnp2,Fos, Fosb,Gatad2a,Nr4a1,Rfc1*
GO:0044249	0.003420	2.3	cellular biosynthetic process	*Camta1,Cir1,Csrnp2,Eif2s3y,Extl1,Fos,Fosb,Gatad2a,Nr4a1,Rfc1*
GO:1901362	0.007914	2.2	organic cyclic compound	*Camta1,Cir1,Csrnp2,Fos,*

			biosynthetic process	*Fosb,Gatad2a,Nr4a1,Rfc1*
GO:1901576	0.003995	2.2	organic substance biosynthetic process	*Camta1,Cir1,Csrnp2,Eif2s3y,Extl1,Fos,Fosb,Gatad2a,Nr4a1,Rfc1*
GO:0010467	0.004468	2.2	gene expression	*Camta1,Cir1,Csrnp2,Eif2s3y,Fos,Fosb,Gatad2a,Nr4a1,Rfc1,Shh*
GO:0009058	0.004604	2.2	biosynthetic process	*Camta1,Cir1,Csrnp2,Eif2s3y,Extl1,Fos,Fosb,Gatad2a,Nr4a1,Rfc1*
GO:0044707	0.037167	2.0	single-multicellular organism process	*Bves,Dvl3,Edn1,Gadd45g,Shh*
GO:0016070	0.016460	2.0	RNA metabolic process	*Camta1,Cir1,Csrnp2,Fos,Fosb,Gatad2a,Nr4a1,Rfc1*
GO:0016787	0.041444	1.8	hydrolase activity	*9930013L23Rik,Abhd2,Eif2s3y,Mtmr11,Rfc1,Shh*
GO:0090304	0.032597	1.8	nucleic acid metabolic process	*Camta1,Cir1,Csrnp2,Fos,Fosb,Gatad2a,Nr4a1,Rfc1*
GO:0043170	0.022166	1.7	macromolecule metabolic process	*Camta1,Cir1,Csrnp2,Eif2s3y,Extl1,Fos,Fosb,Gatad2a,Nr4a1,Rfc1,Shh*
GO:0006807	0.035802	1.7	nitrogen compound metabolic process	*Camta1,Cir1,Csrnp2,Extl1,Fos,Fosb,Gatad2a,Nr4a1,*

				Rfc1
GO:0044260	0.031639	1.7	cellular macromolecule metabolic process	*Camta1,Cir1,Csrnp2,Eif2s3y,Extl1,Fos,Fosb,Gatad2a,Nr4a1,Rfc1*
GO:0050789	0.044778	1.6	regulation of biological process	*Camta1,Cir1,Csrnp2,Dvl3,Edn1,Fos,Fosb,Gatad2a,Nr4a1,Rfc1*
GO:0044237	0.045894	1.5	cellular metabolic process	*Camta1,Cir1,Csrnp2,Eif2s3y,Extl1,Fos,Fosb,Gatad2a,Mtmr11,Nr4a1,Prkacb,Rfc1*

Table S3. Primers for qRT-PCR.

mRNAs	forward primer	reverse primer
Nfkbia	5’- GAAGCCGCTGACCATGGAA -3’	5’- GATCACAGCCAAGTGGAGTGGA-3’
Il6	5’- CCACTTCACAAGTCGGAGGCTTA-3’	5’- CCAGTTTGGTAGCATCCATCATTTC-3’
Rela	5’- GTATTGCTGTGCCTACCCGAAAC-3’	5’- GTTTGAGATCTGCCCTGATGGTAA-3’
Casp3	5’ -CTGCCGGAGTCTGACTGGAA-3’	5’ -ATCAGTCCCACTGTCTGTCTCAATG-3’
Fos	5’- TTACGCCAGAGCGGGAATG-3’	5’- GTTCCCTTCGGATTCTCCGTTT-3’
Mapk1	5’-AACGTTCTGCACCGTGACCTC-3’	5’ -ACCAACGTGTGGCTACGTACTCTG-3’
Mapk3	5’-CTGGACCAGCTCAACCACATTC-3’	5’-AGGTAGTTTCGGGCCTTCATGTTA-3’
Mapk10	5’-ACTCCGGTTTGGAATGTGGCTA-3’	5’-TGCGATGATCCTGCTGGTG-3’
Jun	5’-ATCCACGGCCAACATGCTC-3’	5’- ACGTTTGCAACTGCTGCGTTAG-3’
Tnf	5’-TATGGCCCAGACCCTCACA-3’	5’-GGAGTAGACAAGGTACAACCCATC-3’
Akt1	5’-CATCGTGTGGCAGGATGTGTA-3’	5’ -ACCTGGTGTCAGTCTCAGAGGTG-3’
Akt2	5’-GAAGTCGCCCACACAGTCACA-3’	5’-TTGGCATACTCCATCACAAAGCA-3’
Fis1	5’-TGGGCAACTACCGGCTCAA-3’	5’-TTATCAATCAGGCGTTCCAGCTC-3’
Mfn1	5’-CACTGCAATCTTCGGCCAGTTA-3’	5’-TTTCTGTAGCCCTGTATTTCCACCA-3’
Mfn2	5’-CTCCAAGTGTCCGCTCCTGAA-3’	5’-AGCTGTCCAGCTCCGTGGTA-3’
Dnm1l	5’-CCTCAGATCGTCGTAGTGGGAAC-3’	5’-TGAAACGTGGACTAGCTGCAGAA-3’
Opa1	5’-GCAGCATTAAGACATGAAATCGAAC-3’	5’-CCAGGGCCTTTGACATTTAGAGA-3’
Hmgb1	5’-TCACAGCCATTGCAGTACATTGAG-3’	5’-CCGGCAAGTTTGCACAAAGA-3’
